# Incidence, temporal trend and factors associated with ventilator-associated pneumonia in mainland China: a systematic review and meta-analysis

**DOI:** 10.1186/s12879-017-2566-7

**Published:** 2017-07-04

**Authors:** Chengyi Ding, Yuelun Zhang, Zhirong Yang, Jing Wang, Aoming Jin, Weiwei Wang, Ru Chen, Siyan Zhan

**Affiliations:** 1 Department of Epidemiology and Biostatistics, School of Public Health, Peking University, 38 Xueyuan Road, Haidian District, Beijing, 100191 People’s Republic of China; 2 Division of Epidemiology, The Jockey Club School of Public Health and Primary Care, The Chinese University of Hong Kong, Hong Kong, People’s Republic of China; 3 Primary Care Unit, Department of Public Health and Primary Care, University of Cambridge, Cambridgeshire, UK

**Keywords:** Ventilator-associated pneumonia, Incidence, Risk factors, Meta-analysis, China

## Abstract

**Background:**

Data to date is far from sufficient to describe the recent epidemiology of ventilator-associated pneumonia (VAP) in mainland China. This study aimed to estimate the overall incidence of VAP, with a special focus on its temporal trend and associated factors.

**Methods:**

Meta-analyses of 195 studies published from 2010 to 2015 were conducted, followed by subgroup analyses by methodological quality, pre-defined setting characteristics and attributes of populations.

**Results:**

The overall cumulative VAP incidence in mainland China was 23.8% (95% confidence interval (CI) 20.6–27.2%), with the results showing high heterogeneity. The pooled incidence densities were 24.14 (95% CI 21.19–27.51) episodes and 22.83 (95% CI 19.88–26.23) patients per 1000 ventilator-days. A decline in the cumulative incidence was observed from 2006 (49.5%, 95% CI 40.0–59.0%) to 2014 (19.6%, 95% CI 10.4–31.0%); differences in the incidence rates were also documented according to Chinese provinces and diagnostic criteria (*p* < 0.001). Older age (≥60 years), coma, re-intubation, tracheotomy and prolonged ventilation were the factors significantly associated with the occurrence of VAP.

**Conclusions:**

The incidence of VAP remains high in mainland China but has decreased since 2006. The reported rates vary considerably across individual studies, probably due to variations in diagnosis and geographical region. More studies using standard definitions and cut-off points are needed to better clarify the epidemiology of VAP across the country.

**Electronic supplementary material:**

The online version of this article (doi:10.1186/s12879-017-2566-7) contains supplementary material, which is available to authorized users.

## Background

Ventilator-associated pneumonia (VAP) is the most frequent type of healthcare-associated infection (HCAI) diagnosed in developing countries and has been firmly associated with an increased mortality, a longer hospital stay and additional healthcare costs [1, 2]. However, the incidence of VAP has been defined somewhat differently from study to study; there is currently no consensus on a common numerator or denominator that represents the overall incidence of VAP on a uniform basis. Commonly reported estimates include the percentage of patients on mechanical ventilation who develop VAP (cumulative incidence) and the number of VAP episodes per 1000 ventilator-days and VAP patients per 1000 ventilator-days [3, 4].

During the past few decades, nationwide studies on the incidence of VAP have been rare in China; only three studies have calculated the incidence of VAP at a national level, including one systematic review [5–7]. Based on two multi-center studies that were both conducted from 2013 to 2014, the point estimate of the VAP incidence density in intensive care units (ICUs) was 8.89 patients per 1000 ventilator-days and ranged from 4.50–32.79 patients per 1000 ventilator-days depending on the type of ICU [5, 7]. The systematic review, which included 178 studies published between 2007 and 2012, yielded a pooled VAP cumulative incidence of 33.7% in ICUs [6]. Unfortunately, data regarding the nationwide incidence of VAP outside of ICUs remain unavailable. Moreover, information on the temporal change of the VAP incidence and stratified estimates according to the characteristics of different settings (such as by Chinese province and diagnostic criteria) to date are far from sufficient to describe the epidemiology of VAP across the country.

The main risk factors for VAP, such as prolonged ventilation, pre-existing pulmonary disease, transfusion, re-intubation and enteral feeding, have been extensively described among patients receiving mechanical ventilation in mainland China [8, 9]. However, the reported risk factors were varied or discordant, and there has been no meta-analysis study on this topic until now.

Reliable and updated data on the epidemiology of VAP are needed to inform patients and clinicians, plan healthcare services and policies, ascertain the overall burden of VAP and understand its causes. Given the paucity of existing data, this systematic review aimed to estimate the incidence of VAP in mainland China as well as its temporal trend and associated factors.

## Methods

### Search strategy and selection criteria

This systematic review and meta-analysis followed the Preferred Reporting Items for Systematic reviews and Meta-analyses (PRISMA) statement [10]. We systematically searched five electronic databases including Medline, Embase, the Chinese BioMedical Database (CBM), the China National Knowledge Infrastructure (CNKI) and the Wanfang Database for relevant studies. Given the focus on the recent epidemiology of VAP in our review, these searches were limited to studies published between January 2010 and December 2015. MeSH and free-text terms were applied to Medline, Embase and the CBM, and free-text terms were used to search the CNKI and Wanfang databases. Detailed search strategies for each database are listed in Additional file 1: Table S1. We also hand searched reference lists of relevant studies. The reviewers were divided into two groups that worked in parallel. The titles, keywords and abstracts of each record were screened independently by reviewers according to the eligibility criteria. Potentially eligible studies were further reviewed in their entirety. Disagreements were resolved by consensus.

We placed no restrictions on patient age or diagnostic criteria of pneumonia, and included eligible studies that satisfied all following criteria:

- Involved patients with VAP defined by a joint committee of the American Thoracic Society (ATS) and the Infectious Diseases Society of America (IDSA) as “pneumonia that arises more than 48–72 hours after endotracheal intubation” [11].
- Provided sufficient data to calculate the incidence of VAP. Given the variation in the definition regarding the VAP incidence in different studies and institutions, the following incidences (one cumulative incidence and two incidence densities) were calculated for each study included in our review where applicable: the number of patients with VAP/total number of observed patients on ventilation, VAP episodes per 1000 ventilator-days and VAP patients per 1000 ventilator-days.
- Collected data in a prospective way using a surveillance study design, a cohort study or a nested case control study.
- Were conducted in mainland China. Studies dealing with the VAP incidence in Taiwan, Hong Kong and Macao in China were excluded since the socioeconomic status and healthcare policies in these regions differ from those in mainland China.
- Were published in Chinese or English.

### Assessment of risk of bias

We evaluated the methodological quality of each included study using the modified Leboeuf-Yde and Lauritsen tool [12], which comprise 10 items that measure two study dimensions (external validity and internal validity) plus a summary risk of bias assessment (Additional file 2). Each item can be judged as having either a low or a high risk of bias. One point was awarded for an item if it was considered to have a low risk of bias, and the maximum possible score was 10. Scores of 8 or more, 6–7, and 5 or less were defined as having a low, moderate and high risk of bias, respectively. Graphs of the summary of risk of bias were drawn using Revman 5.3 (Cochrane’s Informatics and Knowledge Management Department, London, UK).

### Data extraction

An extraction form was pre-designed with EpiData 3.1 (The EpiData Association, Odense, Denmark) and then modified based on the results of a pilot test. The revised extraction form consisted of four parts: study setting, methodological quality, characteristics of the populations and data for calculating the incidences of VAP. Two groups of reviewers independently performed the data extraction, and any disagreement was resolved using consensus as well. If possible, the corresponding authors of the included studies were contacted regarding unreported data.

### Statistical analysis

All statistical analyses were carried out using R 3.2.1 (Bell Laboratories, Inc., Madison, WI, USA), and all *p*-values were two-tailed. Heterogeneity was assessed via Q test and *I* ^2^ statistics. *p* < 0.1 or *I* ^2^ > 50% was defined to note substantial heterogeneity [13]. The pooled estimates together with the 95% confidence intervals (CIs) of VAP incidences were obtained using a DerSimonian-Laird random-effects model to accommodate heterogeneity across all included studies [14]. To normalize the distribution of incidence, we implemented an arcsine-transformation for cumulative incidences, while a log-transformation was used to determine the incidence densities. For the log-transformation, increments of 0.5 were added to both the numerators and the denominators in studies with zero reported events. Sensitivity analyses were also conducted by omitting studies that had diagnosed VAP using unclear criteria or in those with a high risk of bias. In addition, the publication bias was examined using Egger’s test [15]. The results were considered to have a probable publication bias when *p* < 0.1.

Furthermore, subgroup analyses were performed using methodological quality and pre-defined setting characteristics, including clinical department, diagnostic criteria, level of hospital, Chinese province and year of study (during which the patients are followed up and the incidence data are collected). Based on the Hospital Grade Management Regulations set by the Chinese Ministry of Health (MoH), hospitals were categorized into tertiary (>500 beds) or non-tertiary (≤500 beds) hospitals according to their size, clinical departments and healthcare personnel. When the incidence was reported for a multi-year period, the midpoint of the time interval was regarded as the year of the study [16, 17]. A line chart was created to graphically demonstrate the temporal trend of the VAP incidence. Additionally, MapInfo Professional 11.0 (Pitney Bowes Inc., Stamford, CT, US) was used to develop a map based on the subgroup analysis by province.

We also conducted subgroup analyses using the attributes of populations. Patient subgroups that were mentioned at least four times in the included studies and had been stratified using comparable cut-off points were screened to carry out the subgroup analyses regarding the attributes of populations in this review. Moreover, a Q test for heterogeneity was used to compare the incidence across subgroups, and 0.05 was defined as the threshold of the *p*-value for statistical significance [18].

## Results

### General information about the included studies

**Fig. 1:**
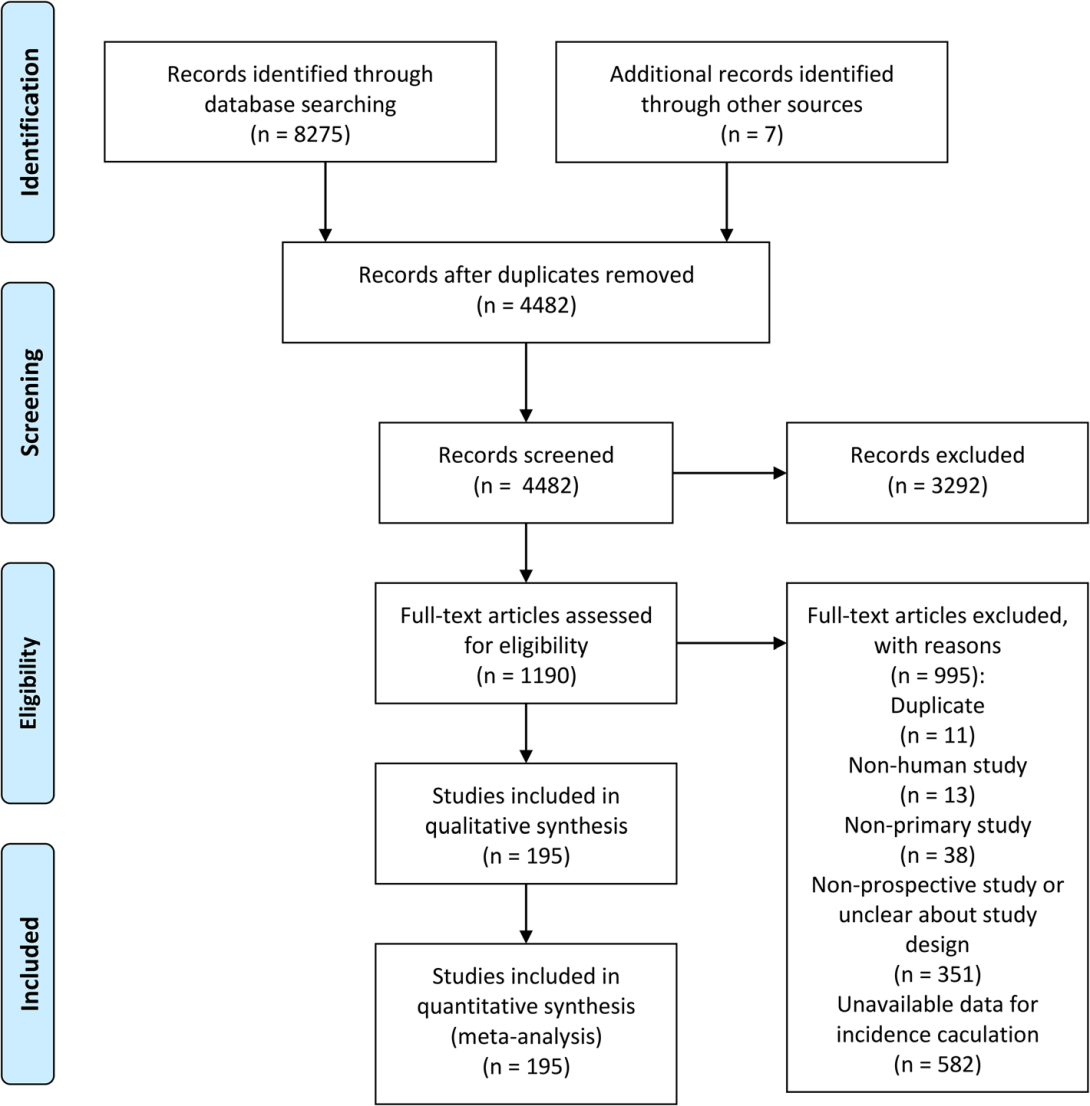
Flow diagram of study inclusion

**Fig. 2:**
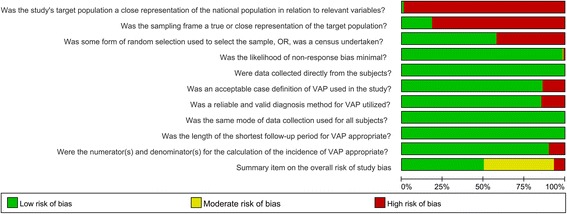
Summary of risk of bias for all the included studies

We identified a total of 8282 records and finally included 195 studies (Fig. 1), of which 93 reported the cumulative incidence of VAP, 50 had an incidence density of VAP episodes per 1000 ventilator-days and 103 reported an estimate of VAP patients per 1000 ventilator-days. The methodological quality of the included studies is illustrated in Fig. 2. Overall, most of the studies demonstrated a good or moderate methodology. A list of the included studies and a summary of their characteristics are presented in Additional file 1: Tables S2 and S3.

### Overall incidence

The pooled cumulative incidence of VAP in mainland China was 23.8% (95% CI 20.6–27.2%) from 2006 to 2014 (Additional file 1: Figure S1). At the same time, the pooled incidence densities were 24.14 (95% CI 21.19–27.51) episodes and 22.83 (95% CI 19.88–26.23) patients per 1000 ventilator-days (Additional file 1: Figure S2 and S3). The Egger’s test indicated that a publication bias was present when verifying the included studies that reported a cumulative incidence or an incidence density of VAP episodes per 1000 ventilator-days (*p* < 0.1 for both estimates).

### Temporal trend in incidence rates

**Fig. 3:**
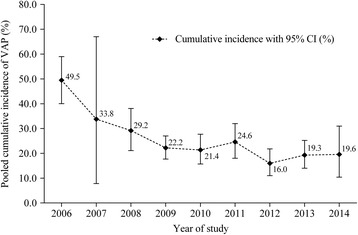
Pooled cumulative incidence of VAP in mainland China at different study periods. Random-effects model was used to pool the individual cumulative incidence of each included study

Because stratified estimates were available in most of the included studies that reported a cumulative incidence, we used the cumulative incidence to conduct all subgroup analyses. The results of the subgroup analyses by methodological quality and study settings are summarized in Table 1 and Additional file 1: Table S4. A declining trend was observed in the cumulative VAP incidence (*p* < 0.001); the pooled estimate in 2006 was 49.5% (95% CI 40.0–59.0%), which had decreased to 19.6% (95% CI 10.4–31.0%) by 2014 (Fig. 3).

**Table 1:** The results of subgroup analyses by methodological quality and pre-defined setting characteristics

Subgroups		Studies	Sample size	Estimate (%)^a^	95% CI (%)	*I* ^*2*^ (%)	*p* value^b^
Overall^c^		93	71,816	23.8	20.6–27.2	98.9	-
Clinical department	General ICU	11	5388	18.3	11.3–26.6	97.7	0.310
	Neonatal ward	4	136	26.1	9.4–47.6	85.5	
	Neonatal ICU	7	3938	15.7	3.2–35.0	99.4	
	Pediatric ICU	6	721	30.2	18.7–43.2	91.8	
	Respiratory ICU	5	607	27.4	23.3–31.7	15.2	
	Surgical ICU	1	85	29.4	20.3–39.5	-	
Diagnostic criteria^d^	By Chinese Critical Care Medicine Society in 2013 ^26^	3	174	38.4	31.4–45.8	0.0	<0.001
	By the Chinese Ministry of Health in 2001 ^25^	29	46,894	20.0	15.1–25.3	99.1	
	By Chinese Thoracic Society in 1999 ^27^	35	13,979	27.2	22.2–32.5	97.6	
Hospital level	Tertiary	75	58,704	23.6	19.7–27.6	99.0	0.574
	Non-tertiary	16	2703	26.4	17.7–36.2	96.3	
Risk of bias	High	9	1286	26.3	17.1–36.6	88.2	0.687
	Moderate	43	17,973	24.6	20.8–28.5	96.8	
	Low	41	52,557	22.2	17.4–27.4	99.2	

*CI* confidence interval, *ICU* intensive care unit, *VAP* ventilator-associated pneumonia

^a^Pooled estimates were calculated by a random-effects model

^b^The Q test for heterogeneity was used to compare the incidence across subgroups

^c^Studies that reported the cumulative incidence of VAP

^d^A summary of diagnostic requirements for the three published sets of criteria is presented in Additional file 1: Table S5

### Incidence rates by geographic region and diagnostic criteria

**Fig. 4:**
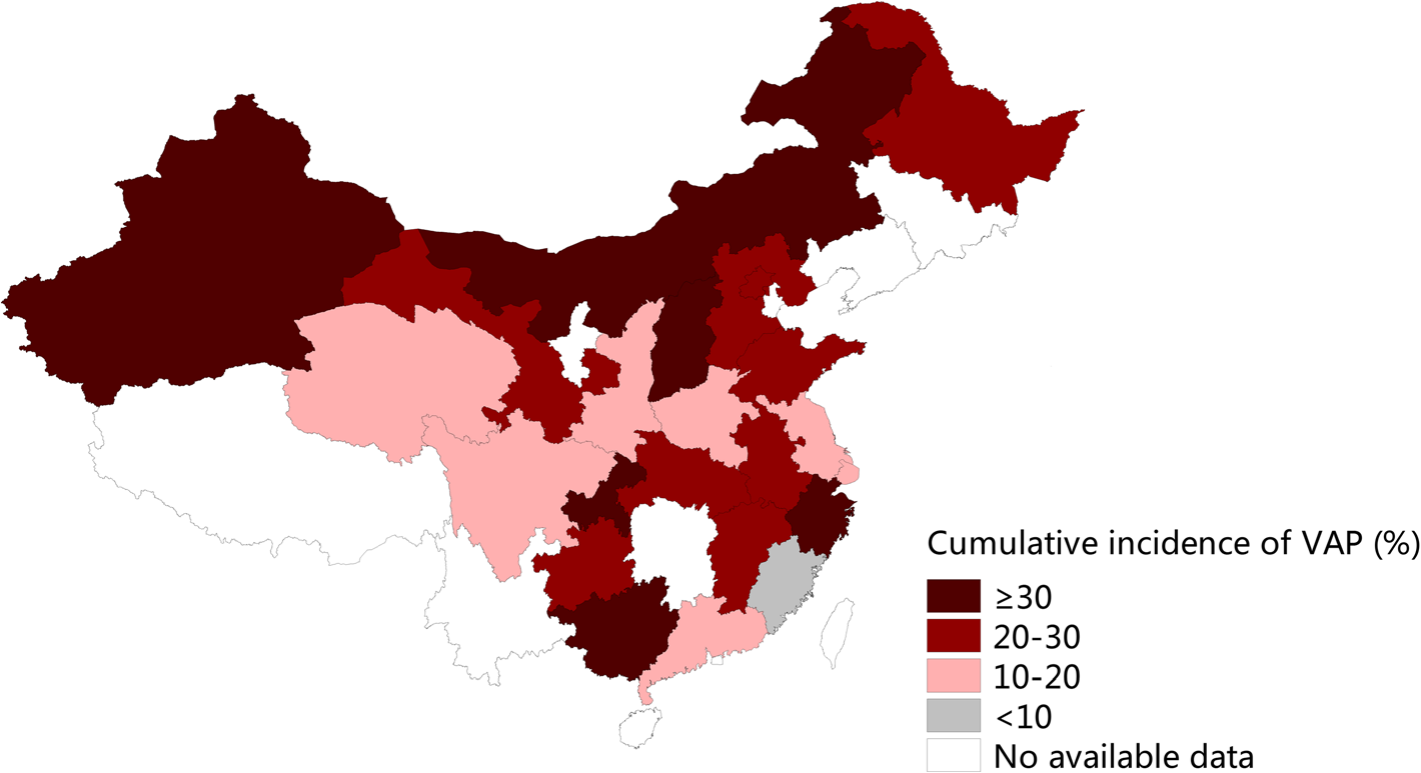
Geographic map showing cumulative incidence of VAP in different Chinese provinces. Map was generated using Mapinfo Professional 11.0 software based on the subgroup analysis by province. Random-effects model was used to pool the provincial cumulative incidence of VAP

The cumulative incidence of VAP varied by geographic region (Fig. 4), with estimates that ranged from 7.5% (95% CI 5.9–9.4%) in the Fujian Province to 70.0% (95% CI 52.7–84.8%) in the Chongqing Province (Additional file 1: Table S4). Differences in incidence rates were also documented according to diagnostic algorithms (*p* < 0.001); the rate ranged from 20.0% (95% CI 15.1–25.3%) to 38.4% (95% CI 31.4–45.8%) when using the three sets of criteria published in Chinese (Table 1, Additional file 1: Table S5) [19–21].

### Factors associated with the development of VAP

Table 2 displays the results of subgroup analyses based on the attributes of populations. The results indicated that VAP occurred more frequently in patients who received a tracheotomy, were re-intubated and were mechanically ventilated for more than two weeks. In addition, older (≥60 years of age) and comatose patients were at greater risk of developing VAP. The cumulative incidence of VAP was higher in men and in patients with diabetes, but the difference was not statistically significant.

**Table 2:** The results of subgroup analyses by characteristics of patient populations

Subgroups	Studies	Sample size	Estimate (%)^a^	95% CI (%)	*I* ^2^ (%)	*p* value^b^
Overall^c^	93	71,816	23.8	20.6–27.2	98.9	-
Gender						
Male	11	2303	22.4	16.6–28.7	90.5	0.085
Female	11	1577	15.8	11.7–20.5	76.8	
Age						
<60 years	4	1219	14.0	10.5–17.9	55.6	0.039
≥60 years	6	956	30.8	15.2–49.1	96.7	
Comatose						
Yes	4	318	55.9	42.5–69.0	79.8	<0.001
No	4	2073	10.2	4.7–17.5	94.6	
Diabetes						
Yes	4	73	42.7	16.4–71.4	84.6	0.276
No	4	442	26.8	20.8–33.2	52.5	
Duration of ventilation						
< 7 days	10	3582	6.6	4.7–8.9	77.7	<0.001
7–13 days	2	208	41.3	34.8–48.1	0.0	
≥14 days	4	114	81.5	57.9–96.7	81.8	
Tracheotomy						
Yes	12	663	55.6	38.2–72.4	94.5	0.002
No	5	448	25.0	17.6–33.2	71.8	
Re-intubation						
Yes	4	209	54.6	43.9–65.2	45.9	<0.001
No	4	1233	15.7	9.9–22.5	82.4	

*CI* confidence interval

^a^Pooled estimates were calculated by a random-effects model

^b^The Q test for heterogeneity was used to compare the incidence across subgroups

^c^Studies that reported the cumulative incidence of ventilator-associated pneumonia

### Sensitivity analyses

In our sensitivity analyses, which excluded studies with unclear diagnostic criteria for VAP, the pooled estimates were 24.1% (95% CI 20.6–27.8%) and 23.46 (95% CI 20.53–26.81) episodes or 21.20 (95% CI 18.26–24.61) patients per 1000 ventilator-days. Sensitivity analyses were also performed by omitting studies with a high risk of bias, which resulted in pooled estimates of 23.6% (95% CI 20.2–27.2%) and 24.30 (95% CI 21.29–27.72) episodes or 22.61 (95% CI 19.57–26.12) patients per 1000 ventilator-days.

## Discussion

In the present study, the pooled cumulative incidence of VAP in mainland China was 23.8%; the incidence density was 24.14 episodes or 22.83 patients per 1000 ventilator-days. These data appear inconsistent with previous findings obtained from two multi-center studies (7.23–8.89 patients per 1000 ventilator-days) or a systematic review (33.7%) on the nationwide VAP incidence [5–7]. The following reasons may have contributed to this inconsistency. First, the incidence of VAP varied by study setting, such as by diagnostic criteria, study period and geographic region. Therefore, variations in these settings may partly account for the differences across the estimated incidences. Second, the present meta-analysis covered approximately 71,816 patients with 1.33 million ventilator-days in mainland China. The sample sizes in our review were substantially larger than those of previous studies (35,903 patients and 101,128 total ventilator-days). A more reliable estimate could be obtained by pooling data with a larger sample size [22]. In addition, our review investigated the incidence of VAP according to characteristics of populations and thus could provide additional useful information.

Our review has shown that VAP represents a critical safety issue for hospitalized patients in mainland China. Compared with the incidence of VAP in developed countries (2.9–7.9 episodes per 1000 ventilator-days) [2, 23], the pooled incidence in mainland China was strikingly higher. The great burden of VAP in mainland China could be the result of a combination of numerous adverse factors, such as understaffing, overcrowding and a lack of knowledge and application of basic infection control measures [2]. For example, although recognized as one of the most cost-effective measures to prevent HCAI, including VAP [24], hand hygiene is very often neglected by healthcare workers in Chinese hospitals, with a suboptimal compliance of 10.6–64.6% that varies depending on clinical department [25].

ICU beds are in short supply in resource-limited countries like China; as a result, many patients who require mechanical ventilation are hospitalized in general wards. For example, the ratio of ICU beds to general beds is 3.5% in a 1500-bed tertiary university-affiliated hospital in Beijing, whereas the figure in the United States is 5–7% [26]. Moreover, data from the same hospital showed that 24.7% of its ventilator-days during 2015–2016 occurred in general wards [27]. Despite the high percentage of patients ventilated in general wards in mainland China, studies regarding the clinical outcomes of this population remain scarce. In the present review, only four studies dealt with mechanical ventilation outside the ICU, and all focused on neonatal wards. The corresponding subgroup analysis yielded a VAP incidence of 26.1% in neonatal wards, which is much higher than the estimate in the neonatal ICU (15.7%). This finding is in line with studies from other resource-limited countries, demonstrating that ICU settings might provide better monitoring, which is associated with fewer complications and more active ventilator management [26, 28]. Given the shortage of ICU beds and the growing number of patients who need them in mainland China, future studies are needed to identify better triage criteria and alternative patient care strategies.

Our review found that the pooled cumulative incidence of VAP decreased notably from 2006 to 2014. In 2005, China committed to reduce HCAI by signing the pledge of the World Health Organization (WHO)‘s First Global Patient Safety Challenge and establishing a national standard for nosocomial infection surveillance [23, 29]. According to these documents, Chinese hospitals must conduct hospital-wide surveillance of HCAI and hire at least one HCAI-control professional per 200–250 beds; a HCAI management committee is also required to supervise the implementation of these actions [29]. A meta-analysis of 11 before-after studies found that this aggressive surveillance strategy resulted in a relative risk reduction of 37% for VAP and 25% for catheter-related bloodstream infection in ICUs [30]. In addition, another meta-analysis found that the incidence of surgical site infection in mainland China has decreased significantly in recent years [31]. Therefore, we believe that these combined efforts could have contributed to the recorded decline of VAP across the country.

Although a nationwide standard for VAP monitoring has been established for many years, data on its incidence are scarce to non-existent in several Chinese provinces, including Xizang, Yunnan, Hunan and Jilin. Previous studies have suggested that the incidence of HCAI tends to be lower in eastern coastal regions relative to those of the midlands and remote western regions because the eastern coastal areas enjoy stronger economies and more abundant healthcare resources [5, 31]. However, an east-to-west gradient was not noted in our geographic mapping of the cumulative VAP incidence. The cause of these observed geographic variations therefore remains unknown but may be due to differences in diverse environmental exposures associated with lung diseases, the implementation of HCAI control measures or the availability of ventilators.

According to our review, the pooled cumulative incidence of VAP varied from 20.0–38.4% depending on which of the three diagnostic algorithms was applied for its identification. Indeed, the incidence rate calculated using the MoH criteria was much lower than the rates obtained using the other two criteria. This difference could have been because the diagnosis of a VAP episode based on the MoH criteria requires a combination of chest radiography signs, clinical symptoms and microbiological findings, while only clinical and radiological findings must be present in the other two sets of criteria; the incidence of VAP tends to decrease when more stringent criteria are applied [19–21, 32, 33]. It has been argued that microbiological findings are essential to avoid the over-diagnosis of VAP; however, the isolation of pathogens without clinical and radiological signs may simply represent colonization [34]. Thus, the diagnosis of this condition should be based on a set of criteria that includes radiological findings, clinical symptoms and microbiology. In light of the preventable nature of VAP, its incidence has served as an indicator of healthcare quality and also of the effectiveness of VAP-prevention strategies. Nevertheless, the subjectivity and discrepancies of the existing Chinese diagnostic criteria make it possible to manipulate the true incidence of this condition [35]. Therefore, relevant studies and surveillance data on this topic should be interpreted with caution.

Mechanical ventilation is a life-saving supportive intervention for critically ill patients, but it can also result in complications, such as lung injury, diaphragmatic dysfunction and VAP [36]. Since the proper identification of risk factors is essential for establishing effective strategies to prevent VAP, we performed this systematic review to resolve the uncertainties found in previous studies; the present study is the first to analyse factors predicting VAP for patients in mainland China using a meta-analysis. As expected, a considerable proportion of the included studies reported a stratified VAP incidence concerning various attributes of populations; however, the definitions or cut-off points of the grouping factors were inconsistent. As a consequence, we used the same definitions and the most common cut-off points to selectively pool stratified estimates that had been reported in at least four studies. The results of this meta-analysis indicated that advanced age (≥60 years), coma, re-intubation, tracheotomy and prolonged ventilation were the factors most frequently associated with the acquisition of VAP in mainland China.

Elderly patients have been shown to be more vulnerable to HCAI due to immunologic involution, age-associated physiological and anatomical alterations and increasingly severe chronic diseases and malnutrition [37]. Accordingly, based on our findings, VAP occurred more frequently in elderly patients than it did in younger adults. Although comatose patients represent a relatively small number of hospitalized patients, this population often comprises critically ill patients with high mortality risks [38]. Moreover, VAP is a common complication in comatose patients that may lead to a poor prognosis. Previous studies have suggested that the use of prone positioning could lower the risk of developing VAP [39, 40]. However, feasibility and safety issues may present barriers to this technique’s implementation; further studies to examine the prevention of VAP in comatose patients are urgently needed.

In accordance with previous studies [41, 42], our review indicated that re-intubation could be a risk factor for VAP. The most likely mechanism underlying this risk is the aspiration of oropharyngeal or gastric secretions contaminated with potentially pathogenic organisms during the intubation procedure. Aspiration appears to play an important role in the development of VAP; therefore, medical staff members should be warned of the risk of aspiration in these patients and be trained in techniques to reduce its incidence. Several studies have proven that performing a tracheotomy earlier rather than later may decrease the incidence of VAP in patients who require prolonged intubation [43, 44]. The high overall incidence of VAP observed in tracheotomy patients in the present study indicated that the optimal timing for performing a tracheotomy in critical care settings in China must be determined; tracheotomy management should also be improved. However, a lack of sufficient data prevented a further subgroup analysis from examining the timing of the tracheotomy. In addition, the cumulative incidence of VAP in our review was highest in patients who were ventilated for more than two weeks. Those who were ventilated for 7–13 days had a lower rate of occurrence than those treated for more than two weeks, while the VAP incidence was the lowest early in the course of ventilation (<7 days). This trend could be explained by the fact that prolonged ventilation increases the risk of infection due to exposure to humidifiers and ventilator circuits that are an important source of pathogens [45]. In light of these findings, strategies to shorten the duration of ventilation may represent another key to controlling VAP, such as a daily interruption for sedative-drug infusions [46] and the application of weaning protocols [47].

There are several limitations of this study. First, about half of the included studies were of moderate or low methodological quality overall. Despite this shortcoming, the methodological quality was not the main source of heterogeneity according to the subgroup analyses. Sensitivity analyses conducted after omitting studies with a high risk of bias showed robust results as well. Second, most of the included studies were conducted in tertiary hospitals located in big cities and therefore might not be representative of the national situation. However, in mainland China, mechanical ventilators are more readily available in tertiary hospitals [48]; our review covered hospitalized patients from more than 20 provinces at the initiation of mechanical ventilation. The risk of bias in generalizing our findings to the entire country has thus been minimized. Furthermore, although a temporal decline in the cumulative incidence of VAP was noted in our review, this result was likely biased since the midpoint of the time interval was used as the year of the study when the incidence was reported for a multi-year period [16, 17].

## Conclusions

In conclusion, the incidence of VAP remains high in mainland China but has decreased since 2006. The reported rates vary widely across individual studies, which is likely due to discrepancies in diagnosis and also variations due to geographical region. Older age, coma, re-intubation, tracheotomy and prolonged ventilation were associated with an increased risk of VAP; improved management and prevention strategies that target these factors should be developed to decrease the incidence rate. In addition, more studies with standard definitions and cut-off points will be required to gain a greater understanding of the epidemiology of VAP nationwide and to provide further reliable evidence in this field.

## Additional files

Additional file 1: **Table S1.** Search strategies and results. **Table S2.** List of all the included studies. **Table S3.** General information of all the included studies. **Table S4.** The results of subgroup analyses by Chinese provinces and years of study. **Table S5.** Summary of diagnostic requirements for the three published sets of criteria in mainland China. **Figure S1.** Forest plot of the cumulative incidence of ventilator-associated pneumonia using a random-effects model. **Figure S2.** Forest plot of the incidence density (reported as episodes per 1000 ventilator-days) of ventilator-associated pneumonia using a random-effects model. **Figure S3.** Forest plot of the incidence density (reported as patients per 1000 ventilator-days) of ventilator-associated pneumonia using a random-effects model. (PDF 2137 kb)

Additional file 2: Risk of bias assessment tool. (PDF 119 kb)

## Abbreviations

ATS: American Thoracic Society
CBM: Chinese BioMedical Database
CI: confidence interval
CNKI: China National Knowledge Infrastructure
HCAI: healthcare-associated infection
ICU: intensive care unit
IDSA: Infectious Diseases Society of America
MoH: Chinese Ministry of Health
PRISMA: Preferred Reporting Items for Systematic reviews and Meta-analyses
VAP: ventilator-associated pneumonia
WHO: World Health Organization
