# Supplementary material for: Incidence, temporal trend and factors associated with ventilator-associated pneumonia in mainland China: a systematic review and meta-analysis
Source: BMC Infect Dis. 2017 Jul 4;17:468. doi: 10.1186/s12879-017-2566-7 (PMC5496595; doi:10.1186/s12879-017-2566-7)
Supplement: Supplementary file 1 — Table S1. Search strategies and results. Table S2. List of all the included studies. Table S3. General information of all the included studies. Table S4. The results of subgroup analyses by Chinese provinces and years of study. Table S5. Summary of diagnostic requirements for the three published sets of criteria in mainland China. Figure S1. Forest plot of the cumulative incidence of ventilator-associated pneumonia using a random-effects model. Figure S2. Forest plot of the incidence density (reported as episodes per 1000 ventilator-days) of ventilator-associated pneumonia using a random-effects model. Figure S3. Forest plot of the incidence density (reported as patients per 1000 ventilator-days) of ventilator-associated pneumonia using a random-effects model. (PDF 2137 kb) [file 12879_2017_2566_MOESM1_ESM.pdf]

Additional file 1: Supplementary tables and figures

Table S1. Search strategies and results

Database	Search stratigies	Restriction to recent years (2010-2015)
Wanfang	((主题:("肺炎"))* ((主题:("前瞻"+"监测"+"随访"+"巢式病例对照"+"套叠式病例对照研究"+"队列")) * (主题:("发病"+"发生"))))	2915
CNKI	(主题="肺炎") and (主题="前瞻"+"监测"+"随访"+"巢式病例对照"+"套叠式病例对照研究"+"队列") and (主题="发病"+"发生")	2437
CBM	("前瞻"+"监测"+"随访"+"巢式病例对照"+"套叠式病例对照研究"+"队列") and ("发生"+"发病") and ("肺炎") [常用字段:智能]	2800
Medline OVID	((surveillance.mp. or exp surveillance/) or (prospective.mp. or exp prospective/) or (follow-up.mp. or exp follow-up/) or (cohort.mp. or exp cohort/) or (nested case-control.mp. or exp nested case-control/)) and ((Pneumonia.mp. or exp Pneumonia/) or (pneumon*.tw.)) and ((China.mp. or exp China/) or (Chinese.mp. or Chinese.tw.)) and (incidence.mp. or exp incidence/)	39
Embase OVID	((surveillance.mp. or exp surveillance/) or (prospective.mp. or exp prospective/) or (follow-up.mp. or exp follow-up/) or (cohort.mp. or exp cohort/) or (nested case-control.mp. or exp nested case-control/)) and ((Pneumonia.mp. or exp Pneumonia/) or (pneumon*.tw.)) and ((China.mp. or exp China/) or (Chinese.mp. or exp Chinese/ or exp Han Chinese/)) and (incidence.mp. or exp incidence/)	84
Total		8275

Table S2. List of all the included studies

No.	ID	Reference
1	Cai 2010	Cai XD, Cao Y, Chen C, Yang Y, Wang CQ, Zhang L, et al. [Investigation of nosocomial infection in the neonatal intensive care unit]. Chinese Journal of Contemporary Pediatrics. 2010; 12(2):81-84.
2	Cai 2015	Cai JP. [Hospital Infection Status and Prevention Measures in an ICU]. Chinese Rural Health Service Administration. 2015; 35(12):1523-1525.
3	Chen 2010a	Chen HP. [Prevalence study and innuence fIactors of nosocomial infection in ICU of the hospital in Jincheng anthracite mining group]. Unpublished Master Dissertation. Shanxi Medical School, 2010.
4	Chen 2010b	Chen T, Liu Y, He Y, Qin GY, Chen M. [The impact of invasive procedure on hospital infections in patients in ICU]. Chinese General Practitioner. 2010; 13(32):3686-3687, 3691.
5	Chen 2010c	Chen XW, Luo XL, Xu SJ, Huang XY, Zhu YR. [Influencing Factors for Ventilator-associated Pneumonia in Pediatric ICU: A Logistic Regression Study and Distribution of Pathogens]. Chinese Journal of Nosocomiology. 2010; 20(21):3270-3272.
6	Chen 2011a	Chen GL, Wu HL, He YQ, Yu JW. [Distribution of ventilator-associated pneumonia pathogens in intensive care unit and drug resistance surveillance]. Chinese Journal of Nosocomiology. 2011; 21(6):1244-1246.
7	Chen 2011b	Chen XM. [Surveillance of pathogens from patients with hospital infection and analysis of drug resistance and hospital infection prevention in intensive care unit]. Unpublished Master Dissertation. Guangzhou Medical School, 2011.
8	Chen 2012	Chen FY, Wang ZY, Wang Y, Xiao C, Qiu SX, Wang SG. [Application of hospital infection monitor system in ventilator-associated pneumonia in ICU]. China Medical Herald. 2012; 9(31):50-51, 54.
9	Chen 2013a	Chen CY. [Objective monitoring and evaluation of intervention for pneumonia after anesthesia]. Modern Journal of Integrated Traditional Chinese And Western Medicine. 2013; 22(10):1085-1087.
10	Chen 2013b	Chen XW, Zhang XK, Luo XL. [Results of targeted surveillance of nosocomial infections from 2009 to 2011 and the changing trends]. Chinese Journal of Nosocomiology. 2013; 23(7):1507-1509.
11	Chen 2013c	Chen YH, Luo JQ. [Analysis of neonatal intensive care unit hospital infection targeted monitoring result]. Medical Innovation of China. 2013(12):43-44.
12	Chen 2013d	Chen WZ, Zhao Z, Huang YL, Lan ZX, Liang JW, Liang LC, et al. [Analysis of the pathogenic bacteria of ventilator associated pneumonia and control countermeasures in comprehensive ICU]. Journal of Tropical Medicine. 2013; 13(7):809-812.
13	Chen 2014	Chen LL, Gui XL, Huang SF, Pan XP. [Distribution and drug resistance monitor of ventilator-associated pneumonia pathogens in ICU]. Chinese Journal of Disinfection. 2014; 31(1):33-34, 37.
14	Chen 2015a	Chen DW, Zhuang GH. [Analysis of risk factors and prevention strategies of targeted monitoring in ICU]. Gansu Medical Journal. 2015; 34(2):128-129.
15	Chen 2015b	Chen SM. [Effect of targeted monitoring of ventilator-associated pneumonia in ICU in Huai'an Second People's Hospital]. Chongqing Medicine. 2015; 44(2):274-275.
16	Cheng 2013	Cheng KP, Kong QF, Zhang XJ, Li JW, Wang CX. [The targeted surveillance and analysis of catheter associated infections in ICU]. Chongqing Medicine. 2013; 42(7):793-794.
17	Cui 2012	Cui RX, Shen SM, Jiang LH, Cui PF. [Targeted monitoring of nosocomial infections in ICU]. Chinese Journal of Nosocomiology. 2012; 22(10):2030-2032.
18	Deng 2015	Deng NM, Wang H, Wang J, Huang M. [Observation on prospective monitoring and cluster intervention of nosocomial infection in intensive care unit of hospital]. Chinese Journal of Disinfection. 2015; 32(10):994-996.
19	Ding 2011	Ding XY. [Results and analysis of investations about the nosocomial infections among neonates in ICUs of Xining area]. Journal of High Altitude Medicine. 2011;

		21(4):57-58.
20	Feng 2013	Feng XR, Li XY, Cui EH. [Ventilator-associated pneumonia in RICU and surveillance]. Chinese Journal of Nosocomiology. 2013; 23(22):5415-5416, 5479.
21	Fu 2010	Fu XF, Lu FS, R. H., Gu Q. [Effects on practice preventive measures of catheter-associated nosocomial infection in surgical intensive care unit]. Chinese Journal of Nosocomiology. 2010(12):1698-1699.
22	Fu 2011	Fu XF, Lu F, Shen RH, Gu QQ. [Efficacy of preventive intervention measures of catheter-associated nosocomial infection in intensive care units]. Chinese Journal of Infection Control. 2011; 10(1):22-25, 46.
23	Gao 2011	Gao FM, Zhang JX, Xu YE. [Analysis of targeted surveillance of ventilator-associated pneumonia in intensive care unit]. Medical Innovation of China. 2011; 8(2):30-31.
24	Gao 2014	Gao F, Zou JN, Luo QQ, Huang HY. [Analysis and results of targeted monitoring of hospical infections in general intensive care unit]. Journal of Nursing Science. 2014; 29(3):56-58.
25	Gao 2015a	Gao HL, Meng CJ, Zhou LQ, Wang Q, Xue P. [Effect of airway management on the incidence of ventilator-associated pneumonia]. China Medical Device Information. 2015; 21(1):110-111.
26	Gao 2015b	Gao XD, Hu BJ, Cui YW, Sun W, Shen Y. [A multicenter prospective monitoring on incidences of ventilator-associated pneumonia in 46 hospitals in China]. Chin J Infect Control. 2015; 14(8):540-543.
27	He 2015	He YG. [Application of oxygenation index window as switching for sequential invasive-noninvasive mechanical ventilator in treating ARDS]. Jiangsu Med J. 2015; 41(4):444-446.
28	Hou 2015	Hou ZM, Wu XC, Cheng Y, Yang W. [Targeted monitoring on intensive care unit in a hospital]. Chin J Infect Control. 2015; 14(2):127-129.
29	Hu 2012	Hu ZY, Shen BH, Li LH, Du YQ. [Incidence and pathogens of ventilator-associated pneumonia in a general intensive care unit of a primary hospital]. Chinese Journal of Infection Control. 2012; 11(2):141-142.
30	Huang 2011a	Huang M. [Effectiveness of targeted monitoring with ICU ventilator associated pneumonia]. China Medicine and Pharmacy. 2011; 01(16):21-22.
31	Huang 2011b	Huang QH. [Investigation and control measures of nosocomial infection in intensive care unit]. Journal of Community Medicine. 2011; 09(18):72-73.
32	Huang 2012	Huang B, Xue FY, Xie JZ, Yao JC, Lin XF. [Study of risk factors for ventilator-associated pneumonia in patients in ICU]. Fujian Medical Journal. 2012; 34(1):137-139.
33	Huang 2013	Huang GX, Ban LS, Liao D, Tang YP, Wei R, Mo WG, et al. [Impact of targeted monitoring on incidence of nosocomial infections in pediatric intensive care unit]. Chinese Journal of Nosocomiology. 2013; 23(22):5463-5465.
34	Huang 2014	Huang NN, Zhu MY, Zhang XX, Wu MS, Tan HJ, Liang YM, et al. [Study on the measures that may reduce the incidence of ventilator-associated pneumonia]. Chinese Journal of Disinfection. 2014:1343-1344.
35	Huang 2015a	Huang Y, Han HJ, Ye Y, Chen JF, Jiang SF, Zhao CL. [Application of targeted surveillance of nosocomial infection in general ICU]. Chin J Emerg Resusc Disaster Med. 2015; 10(6):598-600.
36	Huang 2015b	Huang YZ, Shan MZ, Zhao Q, Wang BC, Sun YH. [Effect of risk assessment on reduction of incidence of ventilator-associated pneumonia]. Chin J Nosocomiol. 2015; 25(21):4909-4910, 4967.
37	Jia 2011	Jia HX, Ren JH, Zhao XL, Li LY. [Research of interventions for device-associated infection in surgical intensive care unit]. Chinese Journal of Infection Control. 2011; 10:166-169.
38	Jia 2015	Jia W, Yang LX, Li YT, Xu JL, Dong Y, Zhang HW. [Targeted monitoring of nosocomial infections in intensive care unit of Tumor Hospital Affiliated to Xinjiang Medical University]. Chin J Infect Chemother. 2015; 15(6):517-522.
39	Jiang 2012a	Jiang SF, Zhou J, Meng J, Liu Y. [Analysis and targeted surveillance in general intensive care unit]. Journal of Frontiers of Medicine. 2012; 02(19):50-51.

40	Jiang 2012b	Jiang YH, Zhou H, Li Y, Shen L. [Target surveillance of nosocomial infections in ICU of 140 hospitals from Jiangsu Province 2011]. Chinese Journal of Nosocomiology. 2012; 22(21):4712-4714.
41	Jiang 2015	Jiang SF, Zhou J, Peng ML, Chen JF, Liu T, Dai YF. [Effect of targeted monitoring on prevention of ventilator-associated pneumonia in ICU]. Chin J Nosocomiol. 2015; 25(1):125-126, 138.
42	Kong 2012	Kong SM, Ju FM. [Targeted surveillance of nosocomial infections in ICU in a secondary-care hospital]. Chinese Journal of Misdiagnostics. 2012; 12(7):1620-1621.
43	Lei 2010	Lei SZ, Hu T, Dai PH. [Nursing cares and interventions for neonatal ventilator-associated pneumonia]. Contemporary Medicine. 2010(28):121-122.
44	Li 2011a	Li MJ, Chen CH, Chen XY, Wang XM, Chen LN, Li L, et al. [Surveillance of Bacterial Colonization and Nosocomial Infections in Neonatal Intensive Care Unit]. Journal of Applied Clinical Pediatrics. 2011; 26(10):762-763, 783.
45	Li 2011b	Li GX, Xu XF, Zhang YC, Yuan TW, Liu YY. [Analysis of nosocomial infections in ICU and its interferentiai measures]. Heilongjiang Medical Journal. 2011; 35(4):292-293.
46	Li 2012	Li WH, Deng M. [Surveillance and analysis of ventilator-associated pneumonia in general intensive care unit]. West China Medical Journal. 2012(9):1285-1288.
47	Li 2013a	Li XH, Yang L, Zou AN, Deng H. [Targeted surveillance and intervention in ventilator-associated pneumonia in an intensive care unit]. Chinese Journal of Infection Control. 2013; 12(4):271-273.
48	Li 2013b	Li Y, Sun DY, Jin XL. [Monitoring and analysis of catheter-related infections in ICU]. Chinese Journal of Nosocomiology. 2013; 23(5):996-997, 1035.
49	Li 2014a	Li J. [Dynamic analysis on effect about intervention and targeted surveillance of nosocomial infections in general ICU]. Guangxi Medical Journal. 2014(11):1617-1619.
50	Li 2014b	Li J. [Targeted monitor on healthcare-associated infection in a general intensive care unit]. Chinese Journal of Infection Control. 2014(8):475-478,499.
51	Li 2014c	Li JM. [Analysis and results of targeted monitoring of nosocomial infection in ICU]. Journal of Qiqihar University of Medicine. 2014; 35(13):1896-1897.
52	Li 2015a	Li JW, Wu LF, Yin WJ, Zong ZY, Zhu SC, Zhang H, et al. [Targeted monitoring on healthcare-associated infection in a respiratory intensive care unit of a teaching hospital]. Chin J Infect Control. 2015; 14(10):708-712.
53	Li 2015b	Li LY, Li HS, Guo YH, Fan J, Meng L, Yao H, et al. [Strengthening competence building for preventing and controlling health-care-associated infection, improving healthcare-associated infection management level]. Chin J Infect Control. 2015;14(8):507-512.
54	Li 2015c	Li M, Peng MQ, Chen QX, Xia XY. [The application of cluster intervention strategies on sedation and analgesia in children with mechanical ventilation]. Anhui Medical and Pharmaceutical Journal. 2015;19(11):2231-2233.
55	Li 2015d	Li RJ, Yang XY, Hui JL, Li H, Pan MS, Yang LQ. [Target monitoring of nosocomial infection in ICU]. Anhui Medical and Pharmaceutical Journal. 2015; 19(7):1298-1301.
56	Liao 2013	Liao D, Huang GX, Ban L, Pan XN, Lai XF, Wei QF, et al. [Targeted surveillance of nosocomial infection in neonatal wards]. Chinese Journal of Neonatology. 2013; 28(3):163-166.
57	Lin 2011	Lin J, Liu SJ. [Targeted surveillance of nosocomial infection in comprehensive ICU]. Chinese Journal of Nosocomiology. 2011; 21(3):445-447.
58	Lin 2014	Lin J, Liu SJ. [Target surveillance of ventilator-associated pneumonia in a general intensive care unit]. Modern Preventive Medicine. 2014(24):4498-4499, 4505.
59	Lin 2015	Lin YR, Wei J. [Bundle treatments for patients with pulmonary tuberculosis and respiratory failure]. Chin J TCM WM Crit Care 2015; 22(3):291-294.
60	Liu 2010	Liu WP: [Study on risk factors for ventilator-associated pneumonia caused by pathogens]. Journal of Baotou Medical College. 2010; 26(2):28-29.
61	Liu 2011	Liu QX, Zhang SH, Zhang XY, Lin WZ. [The effects of targeted surveillance on preventing ventilator-associated pneumonia in intensive care unit]. Fujian Medical Journal. 2011; 33(2):68-69.
62	Liu 2013a	Liu WQ. [Target monitoring of nosocomial infection in ICU]. Harbin Medical Journal. 2013; 33(1):46.
63	Liu 2013b	Liu RL, Wei YH. [Targeted Surveillance Results Analysis of Nosocomial Infection in Comprehensive ICU]. Guide of China Medicine. 2013(24):411-412

64	Liu 2014	Liu XX, Huo HQ, Liang JT, Geng HM, Yin SF. [Targeted surveillance of nosocomial infections in ICUs of a general hospital]. Chinese Journal of Nosocomiology. 2014; 24(13):3220-3222.
65	Liu 2015a	Liu J, Qi SY, Liu ZD, Yang SY. [Analysis of target surveillance of nosocomial infections in ICU]. Anhui J Prev Med. 2015; 21(4):273-276.
66	Liu 2015b	Liu WP, Tian YQ, Cao QL, Qin LL, Li ZH. [Targeted surveillance of nosocomial infections in ICUs of 40 hospitals of Inner Mongolia autonomous region in 2014]. Chin J Nosocomiol. 2015; 25(11):2492-2494.
67	Liu 2015c	Liu XP, Chen HP, Yan YT, Wei HJ. [Influence of intervention measure on morbidity of nosocomial infection in ICU]. Chin J Mod Drug Appl. 2015; 9(2):228-230.
68	Luo 2014	Luo JG, Zhu YH, Liu SL, Wang YP. [Targeted surveillance of NSICU]. Chinese Journal of Nosocomiology. 2014(10):2447-2449.
69	Ma 2012a	Ma XH, Deng SY. [Study on the nursing intervention for ventilator-associated pneumonia in intensive care unit]. Journal of Practical Medical Techniques. 2012; 19(1):105-106.
70	Ma 2012b	Ma LP, Ye H. [Monitoring and prevention of ventilator-associated pneumonia in ICU]. Jilin Medical Journal. 2012; 33(31):6863-6865.
71	Mai 2013	Mai JL, Chen H, Lin SY, Deng YL, Fu XL, Xian ZL. [Targeted surveillance and intervention of nosocomial infection in neonatal ward]. Journal of Guangdong Medical College. 2013; 31(4):395-396.
72	Mao 2010	Mao XQ, Tang CL, Yang CQ. [Ventilator-associated pneumonia in intensive care unit:a prospective study of relative risk factors]. Modern Preventive Medicine. 2010; 37(13):2567-2568.
73	Mao 2012	Mao YP, Han FZ, Zhou H, Zheng W, Zhai RX. [Targeted surveillance of catheter-associated infections in ICU]. Targeted surveillance of NSICU. 2012; 22(12):2506-2508.
74	Mi 2011	Mi CR, Zhang YB, Yang L, Li WH, Gu QY, Zhao YJ, et al. [Catheter-associated nosocomial infection in surgical intensive care unit: a 5 year data analysis]. Chinese Journal of Infection Control. 2011; 10(3):178-180,201.
75	Ming 2012	Ming SL, Liao CJ, Zou XM, Zhang HL. [Integrated ICU Hospital Infection Targeted Research]. Medical Innovation of China. 2012; 10(10):150-151.
76	Pan 2013a	Pan JE, Wu YY, Rong YK. [Target monitoring of ventilator-associated pneumonia in neonatal intensive care unit]. China Medicine and Pharmacy. 2013(11):25-26, 32.
77	Pan 2013b	Pan J, Zhao YP. [Target monitoring of nosocomial infection in ICU]. Medical Innovation of China. 2013(31):136-137,138.
78	Peng 2013	Peng R, Xing JL, Zhu WG. [Results and analysis of targeted surveillance of hospital-acquired infections in ICU]. West China Medical Journal. 2013(8):1179-1181.
79	Pu 2011	Pu D, Shu MR, Zhang WD, Tan C, Zong ZY, Liu HH, et al. [Target monitoring of nosocomial infection in ICU]. Medical Journal of West China. 2011; 23(12):2330-2332.
80	Qi 2010a	Qi YF. [Investigation and analysis of related factors of ventitalor-associated pneumonia in ICU]. Chinese Journal of Practical Nursing. 2010; 26(30):64-65.
81	Qi 2010b	Qi CY. [The assessment of bronchial mucosa biopsy and quantitative culture of bacterial in patients muff-drug resistant bacterial associated with invasive ventilation]. Unpulished Master Dissertation. Hebei Medical School, 2010.
82	Qi 2013	Qi SY, Ma XH, Liu ZD, Li L. [Analysis of risk factors for ventilator-associated pneumonia in comprehensive ICU and nursing strategy]. Chinese Journal of Modern Nursing. 2013; 19(21):2531-2533.
83	Qian 2015	Qian LH, Wang ZL, Zhu JR, Cui Z. [Analysis of nosocomial infection objective monitoring in intensive care unit]. Chinese Journal of General Practice. 2015; 13(8):1273-1274, 1343.
84	Qu 2011	Qu F, Wu SZ, Sheng Y, Yao WS. [Analysis of targeted surveillance of nosocomial infections in neurosurgical intensive care unit in 154 hospitals]. Practical Journal of Medicine Pharmacy. 2011; 28(6):542-543.
85	Qu 2015	Qu D, Ren XX, Guo LY, Xu WJ, Liang JX, Han YH, et al. [The characteristics of bacterial biofilm formation in endotracheal tubes in ventilated patients and the relationship between the biofilm and ventilator-associated pneumonia]. Chin Pediatr Emerg Med. 2015; 22(4):237-240.

86	Ren 2012	Ren JH, Jia HX, Feng Q, Wang Y, Li YL. [Characteristics of nosocomial infections in NICU and targeted monitoring]. Chinese Journal of Nosocomiology. 2012; 22(12):2501-2502, 2505.
87	Ren 2015	Ren JH, Yin H, Wu AH, Hu BJ, Zhang XY, Hou TY, et al. [Multicenter study on epidemiology of device-associated infection in neonatal intensive care units]. Chin J Infect Control. 2015; 14(8):530-534.
88	Shen 2010	Shen Y. [Study on target monitoring applied in monitoring ventilator associated pneumonia in intensive care unit]. Nursing and Rehabilitation Journal. 2010; 09(12):1017-1019.
89	Sheng 2015	Sheng B, Ye Y, Li JB. [Targeted surveillance of nosocomial infections in intensive care units in 2013]. Chin J Infect Chemother. 2015; 15(5):443-446.
90	Shu 2015a	Shu DD. [Application of fiberoptic bronchoscopy and conventional sputum suction therapy on prevention of ventilator-associated pneumonia]. Journal of Clinical Pulmonary Medicine. 2015; 20(4):698-701.
91	Shu 2015b	Shu H, Wei H, Li J, Zhang CL, Xu Q. [Clinical practice of multidimensional strategy to reduce neonatal ventilator-associated pneumonia]. J Clin Pediatr. 2015; 33(5):445-449.
92	Situ 2015	Situ MQ, Li ZY, Zheng L. [Targeted monitoring and control of ventilator associate pneumonia]. Chin J Nosocomiol. 2015; 25(7):1558-1564.
93	Song 2013	Song K, Zhao LX, Yang L, Wu LY. [Target surveillance of ventilator-associated pneumonia in intensive care unit]. For All Health. 2013; 7(7):49.
94	Song 2015	Song L. [Target monitoring of hospital infection in ICU]. Journal of Hebei Medical University. 2015; 36(4):457-459.
95	Sun 2015	Sun JH, Xu SC, Wen L, Zhang LJ, Yan HP. [Analysis of the monitoring results of ICU ventilator associated events]. West China Medical Journal. 2015; 30(2):241-242.
96	Tang 2012a	Tang J, Ye HL, Peng JF, Li CZ, Gao H. [Target surveillance and analysis of nosocomial infection in general ICU]. Medical Journal of Chinese People's Health. 2012; 24(6):679.
97	Tang 2012b	Tang SQ, Yu Q, Jiang R, Yan JX, Cao XW. [Comparison of nosocomial infection by objective surveillance in icu]. Chinese Journal of Disinfection. 2012; 29(6):506-508.
98	Tang 2013	Tang YM, Xie LJ, Wu J. [Targeted surveillance of nosocomial infections in NICU]. Chinese Journal of Nosocomiology. 2013; 23(11):2654-2656.
99	Tang 2015	Tang HP, Gao XD, Fan CS, Sun SS, Zhang YW. [Effect of active screening plus integrated intervention on prevention of multidrug-resistant organisms infections in ICU patient]. Chin J Nosocomiol. 2015; 25(11):2484-2486.
100	Wang 2010a	Wang XJ, Jiang RM, Xu YL, Zhang W, Huang JK, Wang YB, et al. [The analysis of the clinical features between survivors and non-survivors with the severe form of new influenza A (H1N1) viral infection]. Chinese Journal of Tuberculosis and Respiratory Diseases. 2010; 33(6):406-410.
101	Wang 2010b	Wang XY. [Pathogens isolated from breathing circuit of patients with mechanical ventilation in general intensive care unit: analysis and intervention]. Unpublished Master Dissertation. Nanfang Medical School, 2010.
102	Wang 2010c	Wang XW, Gu KJ, Zhong XJ, Tao JM, Zhu YF. [Analysis of the pathogens from endotracheal aspirate cultures in patients with ventilator-associated pneumonia]. Clinical Medicine of China. 2010; 26(3):289-291.
103	Wang 2011a	Wang B. [Analysis on Target Monitoring and intervention of Catheter - associated Nosocomial Infection in SICU]. Xinjiang Medical Journal. 2011; 41(3):26-29.
104	Wang 2011b	Wang HM, Wang L, Liu FF. [Investigation of VAP-preventive nursing practice in ICU patients]. Journal of Nursing Science. 2011; 26(13):85-86.
105	Wang 2011c	Wang H. [The value of quantitatiVe culture of bacteria got by PSB combining with bacterial mucosa biopsy in MV patients with multi-drug resistant bacteria]. Unplublished Master Dissertation. Hebei Medical School, 2011.
106	Wang 2012	Wang K. [Clinical analysis of etiology of ventilator-associated pneumonia in CSICU]. Chinese Journal of Nosocomiology. 2012; 22(23):5227-5229.
107	Wang 2013a	Wang AC, Xie M, Liu WL. [Analysis of the target monitoring of nosocomial infection in ICU]. Chinese Journal of Modern Nursing. 2013; 19(29):3673-3676.
108	Wang 2013b	Wang XP, Yang YP. [Target monitoring of nosocomial infections in ICU and analysis of risk factors]. Chinese Journal of Nosocomiology. 2013; 23(15):3594-3596

109 Wang 2013c Wang QH. [A clinical analysis of 59 cases of ventilator-associated pneumonia in intensive care unit]. China Foreign Medical Treatment. 2013(31):21-23.

110 Wang 2013d Wang YL, Yang XH, Wang H, Zhao Z, Yan Y. [Target monitoring of nosocomial infection in ICU]. Medical Information. 2013; 26(30):171-172.

111 Wang 2014a Wang HH. [The prevention and control experience about ventilator-associated pneumonia (VAP) with PDCA]. Journal of Henan Medical College for Staff and Workers. 2014; 26(1):46-48.

112 Wang 2014b Wang L, Sun HZ, Cui XH, Huang JH. [Investigation and analysis of risk factors of ICU-acquired pneumonia]. Journal of Qilu Nursing. 2014(2):67-68.

113 Wang 2014c Wang QQ, Su WD, Zhang WW, Lin ZL. [Targeted surveillance and risk factors on preterm infants with nosocomial infections]. Chinese Journal of Neonatology. 2014; 29(4):233-237.

114 Wang 2015a Wang XB, You RK, Shao ZZ, Chen MH. [Targeted monitoring of nosocomial infections in general ICU]. Chin J Nosocomiol. 2015; 25(1):116-118.

115 Wang 2015b Wang J, Chen MY, Huang J. [Targeted surveillance of nosocomial infections in general ICU]. Chin J Nosocomiol. 2015; 25(10):2242-2243, 2264.

116 Wang 2015c Wang L. [Intervention in healthcare-associated infection in a neonatal intensive care unit]. Chin J Infect Control. 2015; 14(12):830-833.

117 Wang 2015d Wang W. [Target monitoring of ventilator-associated pneumonia in neonatal intensive care unit]. Maternal and Child Health Care of China. 2015; 30(5):721-722.

118 Wei 2010 Wei LY, Ye XZ, Li ZM, Qiu YT. [Targeted monitoring on nosocomial infection in a neonatal intensive care unit]. South China Journal of Preventive Medicine. 2010(2):48-50.

119 Wei 2012 Wei Y, Yu LL, Re YH, Ding LL, Wang P, Gu XT. [Targeted monitoring of nosocomial infections in ICU]. Chinese Journal of Nosocomiology. 2012; 22(19):4199-4201.

120 Wei 2013 Wei Y, Wang P, Ding LL, Yu LL, Gu XT, Ma ZY. [Implications for control of nosocomial infections in intensive care unit]. Chinese Journal of Infection and Chemotherapy. 2013:438-441.

121 Wen 2013 Wen SQ. [Cluster of care preventing ventilator associated pneumonia: case-control study]. Journal of Frontiers of Medicine. 2013(18):39-40

122 Wu 2012 Wu XL, Wu ZJ, Zhang YL, He SL, Zhu YP. [Targeted monitoring on healthcare-associated infection in a neonatal intensive care unit in 2010]. Chinese Journal of Infection Control. 2012; 11(4):278-281.

123 Wu 2013 Wu HM, Xu ZQ, Jin XG, Chen YF, Gao SC, Cheng HJ. [Analysis and target surveillance of ventilator-associated pneumonia in intensive care unit]. Zhejiang Medical Journal. 2013(12):1206-1208.

124 Wu 2015 Wu XP, Wang JP. [Targeted monitoring of nosocomial infections in ICU]. Hebei Medical Journal. 2015; 37(11):1723-1725.

125 Xiang 2014 Xiang XL, Song XY, Zheng XH, Yang QQ. [Targeted Surveillance to Hospital Infection in Intensive Care Unit]. West China Medical Journal. 2014(3):432-435.

126 Xie 2011 Xie JL, Qin Y, Qian QZ, Shi CJ. [Monitoring of influenza A H1N1 nosocomial infection]. Jiangsu Healthcare Administration. 2011; 22(6):80-81.

127 Xie 2014 Xie DS, Ming X, Hu Y, Luo QQ, Fu XY, Wang HF, et al. [Distribution and drug resistance of pathogens causing ventilator-associated pneumonia]. Chinese Journal of Nosocomiology. 2014(21):5245-5246, 5257.

128 Xing 2012 Xing HL, Tian HZ, Yuan ZH, Liu G. [Changes of pathogens distribution and targeted surveillance after the ICU moving into the laminar air flow ward]. Guide of China Medicine. 2012(24):429-431.

129 Xing 2013 Xing HL, Yang J, Yin XL. [Target monitoring in 75 newborns in intensive care unit]. Medical Information. 2013(28):303-303,304.

130 Xu 2010a Xu HM, Wang JM, Qie GL, Liu HT, He JZ. [Risk Factors and Pathogens in Elderly Patients with Ventilator-associated Pneumonia]. Chinese Journal of Nosocomiology. 2010; 20(4):494-496.

131 Xu 2010b Xu Z, Tang SQ, Cao XW, Yu Q, Rao XY. [Targeted Surveillance of Nosocomial Infection in Neonatal Wards]. Chinese Journal of Nosocomiology. 2010; 20(21)3324-3326.

132 Xu 2014a Xu XL, Liang YP, Yang P, Ma WC, Hou C. [Nosocomial infection targeted monitoring analysis and prevention measures in ICU]. Chinese Journal of Nosocomiology. 2014(8):1902-1904.

133 Xu 2014b Xu Y, Wang WJ, Xu RL, Zhou T. [Analysis of Surveillance and Risk Factors of Ventilator-associated Pneumonia in a Pediatric Intensive Care Unit]. Modern Preventive Medicine. 2014(8):1530-1532.
134 Xue 2014 Xue JL, Zhang X, Fan HY. [Analysis of targeted monitoring of nosocomial infections in general ICU]. Chinese Journal of Nosocomiology. 2014; 24(3):627-629.
135 Xue 2015 Xue FG. [Effect of Cluster Intervention Strategies on Ventilator Associated Pneumonia]. Medical Information. 2015; 28(47):107-108.
136 Yan 2014 Yan SZ, Bu GL, Miao L, Wang H. [Analysis of relevant factors for nosocomial infection in ICU]. Chinese Journal of Nosocomiology. 2014(24):6112-6113, 6121.
137 Yang 2012 Yang YH, Lu LH, Wu XF. [Target monitoring and analysis of ventilator-associated pneumonia]. Chinese Journal of Nosocomiology. 2012; 22(15):3245-3247.
138 Yang 2013 Yang SZ, Cheng KP. [Surveillance on healthcare-associated infection in patients in an intensive care unit]. Chinese Journal of Infection Control. 2013; 12(6):466-467, 470.
139 Yang 2014a Yang HN, Wang P, Chen JH, Zhang N. [Net surveillance and intervention study of the ventilator associated pneumonia]. Chinese Journal of Disinfection. 2014; 31(1):50-51, 54.
140 Yang 2014b Yang J, Dong CM, Li JY, Zhang HS, Zhang H, Zhu RX, et al. [Ventilator bundle treatment of acute respiratory distress syndrome and its correlation with biomarkers of inflammation]. Chinese Critical Care Medicine. 2014; 26(8):544-548.
141 Yang 2014c Yang J, Wang HY, Liang H, Xi JJ, Cheng R. [Prospective study of risk factors in ventilator-associated pneumonia in mechanically ventilated patients in ICU]. Chinese Journal of Emergency. 2014; 23(11):1239-1243.
142 Yang 2014d Yang XL, Du XY, Zhou YL. [Analysis of the targeted surveillance of nosocomial infection in intensive care unit and the corresponding management tactics]. Today Nurse. 2014(8):141-142.
143 Yang 2015a Yang XX, Xing YW, Ma M, Chen JZ, Wang Y, Liu Y, et al. [Target monitoring of nosocomial infection in a Tumor Hospital ICU]. Modem Preventive Medicine. 2015; 42(7):1336-1338.
144 Yang 2015b Yang ZY, Shen ZX, Wen MD, Lu YC. [Effect of automatic control of air pressure suction combined with continuous subglottic on ventilator-associated pneumonia]. Chin J Mod Nurs. 2015; 21(20):2466-2468.
145 Yao 2015 Yao LL. Present Situation Investigation and Drug Resistance Analysis of Nosocomial Infection Pathogens in Intensive Care Unit. Unpublished Master Dissertation. Hebei Medical University, 2015.
146 Ye 2014 Ye H, Fan HY, Qian AP, He WF, Chen YH. [Target surverlliance of ventilator-associated pneumonia]. Clinical Education of General Practice. 2014(2):215-217.
147 Ying 2011 Ying RL. [Target Monitoring and Control of Ventilator-Associated Pneumonia in Neonatal Intensive Care Unit]. Chinese General Practitioner. 2011; 14(27):3154-3155.
148 Ying 2013 Ying RL. [Study on the effect of ventilator bundle intervention strategies in prevention of neonatal ventilator-associated pneumonia]. Maternal & Child Health Care of China. 2013; 28(34):5720-5722.
149 You 2012 You WQ, Tang GY: [The effects of non-drug prevention on preventing ventilator-associated pneumonia in ICU]. Medical Innovation of China. 2012; 9(2):122-123.
150 Yu 2011a Yu HT, Jia JG, Wang M, Zang JP, Liu YH. [Analysis of Risk Factors for Ventilator-Associated Pneumonia in Mechanically Ventilated Patients in Respiratory Intensive Care Unit]. Chinese Journal of Respiratory and Critical Care. 2011; 10(1):69-71.
151 Yu 2011b Yu GL, Chen CH, Xu CL, Lin RH, Zhang C. [Target surveillance of ventilator-associated pneumonia in intensive care unit]. Chinese Journal of Nosocomiology. 2011; 21(23):4941-4943.
152 Yu 2012a Yu H, Yang HY, Liu YM. [Risk factors for ventilator-associated pneumonia in an intensive care unit: a prospective study]. Shanxi Medical Journal. 2012; 41(11):1121-1123.
153 Yu 2012b Yu LX, Q. CQ, Wang HP. [Survey on the current status of nosocomial infections in ICU]. Chinese Journal of Disinfection. 2012; 29(1):37-38.
154 Yu 2014 Yu ZR, Liu QH, Xu GF. [Surveillance of ventilator-associated pneumonia in intensive care unit and the nursing interventions]. Medical Information.

2014(32):320-321.

155 Yu 2015a Yu H, Zhu JM, Hu K. [Effect of Bundle Intervention Strategies on the Incidence of Ventilator Associated Pneumonia]. Chinese Journal of Disinfection. 2015; 32(9):939-941.

156 Yu 2015b Yu HH, Shao XP. [Clinical Trial of Oral Care Combined with Tooth Brushing in Preventing Ventilator-associated Pneumonia and Relevant Adverse Events]. Nurs J Chin PLA. 2015; 32(21):39-42.

157 Yu 2015c Yu YH, Ni FY, Shen B. [Effect of target monitoring in the control of ventilator associated pneumonia in ICU]. Jiangsu Healthcare Administration. 2015; 26(5):42-44.

158 Yuan 2011 Yuan XN, Xie XS, Ma XM. [Surveillance and analysis of nosocomial infections in ICU in certain 3A Beijing hospitals]. Chinese Nursing Management. 2011(10):47-49.

159 Yue 2015 Yue MK, Liu FR, Zhao L, Zhang FS, Wang CT. [A multicenter clinical study of bundle treatment for moderate or severe acute respiratory distress syndrome]. Chin Crit Care Med. 2015; 27(7):601-605.

160 Zeng 2014 Zeng CH, He J, Li XM, Zhang CL, Zhong H. [Analysis of clinical outcomes of patients with mechanical ventilation in the intensive care unit]. West China Medical Journal. 2014; 03:461-462.

161 Zhang 2010a Zhang YW. [Effectiveness of bundle for the incidence of Ventilator-associated pneumonia]. Unpublished Master Dissertation. Fudan University, 2010.

162 Zhang 2010b Zhang JG, Peng ZL, Liu Z, Zhang N. [Surveillance and distribution of pathogens isolated from patients with neonatal ventilator-associated pneumonia in Yulin city in 2009]. Guide of China Medicine. 2010; 8(12):108-109.

163 Zhang 2011a Zhang HP, Zhang YQ, Zheng D, Yang YM, Li SG. [Target monitoring of nosocomial infection in general intensive care unit]. Chinese Journal of Nosocomiology. 2011; 21(1):49-51.

164 Zhang 2011b Zhang LY. [Targeted surveillance of ventilator-associated pneumonia in an intensive care unit]. Journal of Pratical Medical Techniques. 2011; 18(1):22.

165 Zhang 2011c Zhang WH, Yi GZ, Yuan Z, Huang WX, Du YP, Su XQ. [Target monitoring of nosocomial infection in intensive care unit]. Chongqing Medicine. 2011; 40(34):3457-3459.

166 Zhang 2011d Zhang WH, Yuan Z, Huang WX, Du YP, Yi GZ, Su XQ. [Analysis and strategies of targeted surveillance for nosocomial infection in ICU ward of pulmonary medicine]. Chongqing Medicine. 2011; 40(36):3657-3659.

167 Zhang 2011e Zhang XQ:.[Targeted surveillance on hospital infection in intensive care unit]. Modern Preventive Medicine. 2011; 38(19):3946-3947, 3957.

168 Zhang 2011f Zhang YQ, Gan MX, Lin X, Xiao ZM, Guo YY. [Targeted surveillance and intervention in reducing ventilator-associated pneumonia]. Chinese Journal of Infection Control. 2011; 10(1):30-32.

169 Zhang 2012a Zhang ZQ. [Mechanical ventilation in patients with ventilator-associated pneumonia risk factors and prognosis]. Unpublished Master Dissertation. Shandong University, 2012.

170 Zhang 2012b Zhang P, Zhou XC, Fan SH. [Analysis of nosocomial infections in an intensive care unit]. Journal of Snake. 2012; 24(2):144-146.

171 Zhang 2013a Zhang WY, Tan J, Yu SY. [Analysis of risk factors of ventilator-associated pneumonia]. Jiangxi Medical Journal. 2013(12):1304-1305.

172 Zhang 2013b Zhang P, Wang GM, Jiao JY, Qin CL, Ge M. [Targeted surveillance on nosocomial infection and analysis on intervention effect in integrated ICU]. Occupation and Health. 2013; 29(3):360-362.

173 Zhang 2014a Zhang XQ, Zhao X. [Targeted monitoring of nosocomial infections in intensive care unit]. Journal of Medical Forum. 2014(3):82-83.

174 Zhang 2014b Zhang Y, Wu ST. [The results of targeted surveillance of nosocomial infections in a neonatal intense care unit]. Chinese Journal of Modern Drug Application. 2014(24):217-218.

175 Zhang 2014c Zhang JY, Lu F, Gu HF. [Analysis of the targeted monitoring of nosocomial infections in neonatal intensive care unit]. Chinese Journal Of Public Health Engineering. 2014(2):157-158, 161.

176	Zhang 2015a	Zhang RY. [Application of the artificial airway gasbag pressure in patients with mechanical ventilation]. China Prac Med. 2015; 10(5):270-271.
177	Zhang 2015b	Zhang SM, Zhu Y, Pan YY, Zhuang JW, Zhang JH. [Research on effects of management modes on reduction of incidence of ventilator-associated pneumonia in ICU patients]. Chin J Nosocomiol. 2015; 25(11):2505-2507.
178	Zhang 2015c	Zhang XX, Zuo ZL. [Clinical research of ε-polylysine in the prevention of ventilator-associated pneumonia for children with mechanical ventilation in PICU]. Chin J Mod Nurs. 2015; 21(3):249-253.
179	Zhao 2012a	Zhao Z, He HM, Shen XP, Tan QF. [Target surveillance of ventilator-associated pneumonia in inpatients during 2009-2011]. Journal of Qiqihar University of Medicine. 2012; 33(13):1783-1785.
180	Zhao 2012b	Zhao N, Dong XB, Hao MM, Zhang Y, Zhao LY. [Targeted surveillance of nosocomial infection in ICU]. Chinese Journal of Aesthetic Medicine. 2012; 21(16):435-436.
181	Zhao 2013	Zhao LH, Chen CF, Chen CY, Zou YC, Kong DB. [Targeted surveillance of nosocomial infections in ICU in 2012]. Chinese Journal of Nosocomiology. 2013; 23(20):4883-4884, 4887.
182	Zhao 2014a	Zhao DY, Guo QZ, Zhang SP. [Distribution and drug resistance of pathogens causing ventilator-associated pneumonia in children]. Chinese Journal of Nosocomiology. 2014; 24(6):1523-1525.
183	Zhao 2014b	Zhao DY, Zhou Y, Situ XM. [Targeted monitoring of nosocomial infections in Pediatric CICU]. Chinese Journal of Nosocomiology. 2014(8):2039-2041.
184	Zhao 2015	Zhao XB. The effect of comprehensive intervention measures on the incidence of ventilator associated pneumonia in ICU. Unpublished Master Dissertation. Hebei Medical University, 2015.
185	Zhong 2013	Zhong Y. [Study on the bundled care program in preventing ventilator-associated pneumonia]. World Health Digest. 2013(23):321-322.
186	Zhou 2013a	Zhou H, Jiang YH, Shen L, Li Y, Zheng W, Han FP, et al. [Targeted surveillance of nosocomial infections in ICUs between grade 2 and grade 3 hospitals: a comparative study]. Chinese Journal of Nosocomiology. 2013; 23(7):1515-1517.
187	Zhou 2013b	Zhou L. [Targeted Surveillance of Nosocomial Infection in Intensive Care Unit]. Chongqing Medicine. 2013; 42(1):69-71.
188	Zhou 2013c	Zhou X, Huang GX, Chen XX, Li YJ, Qu HH. [Targeted surveillance and interventions on nosocomial infection in an intensive care unit]. Journal of Chongqing Medical University. 2013(07):729-732.
189	Zhu 2010	Zhu Q, Yang BZ. [Targeted surveillance of Nosocomial Infection in neurological ICU]. Journal of Ningxia Medical University. 2010; 32(3):384-387.
190	Zhu 2014a	Zhu H, Gu J, Ma J. [Targeted monitoring and interventions in ICU of general hospital]. Chinese Journal of Nosocomiology. 2014(17):4382-4384.
191	Zhu 2014b	Zhu Y, Zhang SM, Pan YY, Zhuang JW. [Effect of targeted monitoring on control of nosocomial infections in department of critical care medicine]. Chinese Journal of Nosocomiology. 2014(16):4133-4135.
192	Zong 2013	Zong YZ, Chen LP, Xu XX, Zou QY, Li HF. [Analysis and improvement of targeted surveillance of nosocomial infections in general ICU]. International Journal of Nursing 2013; 32(9):2132-2134.
193	Zou 2013	Zou JN, Zhu M, Huang HY, Wu YY, Deng M, Xiong LJ. [Surveillance of catheter-related infections in ICU during 2011-2012]. Chinese Journal of Nosocomiology 2013; 23(24):5969-5970, 5973.
194	Zou 2015	Zou WH, Guo YX, Chang YJ, Wei Y, Hu YM, Wu MX. [Target monitoring and nursing strategy of nosocomial infection in PICU]. The World Clinical Medicine. 2015; 9(5):248-249.
195	Zuo 2015	Zuo YS, Luo SH. [Surveillance on healthcare-associated infection in a neonatal intensive care unit of a hospital for three consecutive years]. Chin J Infect Control. 2015; 14(1):45-47.

Table S3. General information of all the included studies

Study ID	Risk of bias	Province	Start	Finishing	Study design	Clinical department[a]	Hospital level	Diagnostic criteria[b]	VAP patients per 1000 ventilator-days	VAP episodes per 1000 ventilator-days	Cumulative incidence
Cai 2010	low	Shanghai	Feb 2006	Jan 2007	Surveillance	NICU	Tertiary	Others		√	
Cai 2015	moderate	Zhejiang	Mar 2013	Jul 2014	Surveillance	ICU	Non-tertiary	Unclear	√		
Chen 2010a	low	Shanxi	Sep 2008	Dec 2009	Surveillance	ICU	Tertiary	Criteria by Chinese Thoracic Society in 1999		√	
Chen 2010b	low	Guizhou	Apr 2009	Mar 2010	Surveillance	ICU	Tertiary	Criteria by the Chinese Ministry of Health in 2001			√
Chen 2010c	low	Jiangxi	Jan 2008	Dec 2008	Surveillance	PICU	Tertiary	Criteria by Chinese Thoracic Society in 1999			√
Chen 2011a	moderate	Jiangxi	Jan 2007	Jan 2008	Surveillance	ICU	Tertiary	Others			√
Chen 2011b	moderate	Guangdong	Jan 2009	Dec 2010	Surveillance	General ICU	Tertiary	Criteria by the Chinese Ministry of Health in 2001		√	
Chen 2012	low	Hubei	Jan 2010	Oct 2011	Surveillance	ICU	Non-tertiary	Criteria by the Chinese Ministry of Health in 2001	√		√
Chen 2013a	low	Hebei	May 2010	May 2011	Surveillance	ICU	Non-tertiary	Criteria by Chinese Thoracic Society in 1999			√
Chen 2013b	low	Jiangxi	Jul 2009	Jun 2011	Surveillance	Unclear	Tertiary and Non-tertiary	Criteria by the Chinese Ministry of Health in 2001	√		√
Chen 2013c	moderate	Guangxi	Mar 2011	Feb 2012	Surveillance	NICU	Tertiary	Criteria by the Chinese Ministry of Health in 2001		√	
Chen 2013d	low	Guangdong	Jan 2009	Dec 2011	Surveillance	General ICU	Non-tertiary	Others			√
Chen 2014	low	Jiangxi	Jan 2010	Dec 2011	Surveillance	ICU	Tertiary	Others			√
Chen 2015a	low	Shaanxi	Sep 2013	Nov 2013	Surveillance	ICU	unclear	Criteria by the Chinese Ministry of Health in 2001		√	
Chen 2015b	low	Jiangsu	Jul 2012	Jun 2013	Surveillance	General ICU	Tertiary and Non-tertiary	Criteria by Chinese Thoracic Society in 1999	√	√	
Cheng 2013	low	Jiangsu	Jan 2011	Dec 2011	Surveillance	ICU	Tertiary	Criteria by the Chinese Ministry of Health in 2001	√		
Cui 2012	low	Jiangsu	Jan 2010	Dec 2010	Surveillance	ICU	Tertiary	Criteria by the Chinese	√		

								Ministry of Health in 2001			
Deng 2015	moderate	Shandong	Jan 2011	Dec 2013	Surveillance	ICU	Tertiary	Criteria by the Chinese Ministry of Health in 2001			√
Ding 2011	low	Qinghai	Jan 2009	Dec 2010	Surveillance	NICU	Tertiary	Criteria by the Chinese Ministry of Health in 2001			√
Feng 2013	moderate	Zhejiang	Jan 2008	Dec 2012	Surveillance	RICU	Tertiary	Criteria by Chinese Thoracic Society in 1999			√
Fu 2010	low	Shanghai	Jan 2006	Dec 2009	Surveillance	SICU	Tertiary	Criteria by the Chinese Ministry of Health in 2001	√		
Fu 2011	low	Shanghai	Jan 2006	Dec 2009	Surveillance	Medical ICU, SICU, Neurosurgery ICU, Cardiac surgery ICU, and Transplantation ICU	Tertiary	Criteria by the Chinese Ministry of Health in 2001	√		
Gao 2011	low	Shanxi	Jul 2008	Jun 2009	Surveillance	ICU	Tertiary	Criteria by the Chinese Ministry of Health in 2001			√
Gao 2014	low	Hubei	Jan 2012	Dec 2012	Surveillance	General ICU	Tertiary	Criteria by the Chinese Ministry of Health in 2001	√	√	
Gao 2015a	moderate	Heilongjiang	Oct 2014	Mar 2015	Prospective cohort study	RICU	Tertiary	Others	√		√
Gao 2015b	low	Unclear	Oct 2013	Sep 2014	Surveillance	General ICU	Tertiary and Non-tertiary	Criteria by Chinese Thoracic Society in 1999	√		
He 2015	high	Zhejiang	Jan 2012	Jan 2014	Prospective cohort study	ICU	Non-tertiary	Unclear	√		√
Hou 2015	low	Chongqing	Jan 2013	Dec 2013	Surveillance	ICU	Tertiary	Criteria by the Chinese Ministry of Health in 2001	√		
Hu 2012	low	Hubei	Jul 2009	Jun 2010	Surveillance	ICU	Tertiary	Criteria by Chinese Thoracic Society in 1999			√
Huang 2011a	moderate	Sichuan	Jan 2009	Dec 2010	Surveillance	ICU	Tertiary	Unclear			√
Huang 2011b	moderate	Sichuan	Jan 2010	Dec 2010	Surveillance	ICU	Non-tertiary	Criteria by the Chinese Ministry of Health in 2001			√
Huang 2012	moderate	Fujian	Jul 2008	Jun 2011	Prospective cohort study	ICU	Tertiary	Criteria by the Chinese Ministry of Health in 2001	√		√
Huang 2013	low	Guangxi	Jan 2011	Jun 2012	Surveillance	PICU	Tertiary	Criteria by the Chinese Ministry of Health in 2001	√		√
Huang 2014	low	Guangxi	Jan 2010	Dec 2013	Surveillance	ICU	Tertiary	Others	√		

Huang 2015a	high	Jiangsu	Jan 2012	Dec 2012	Surveillance	General ICU	Tertiary	Unclear	√		
Huang 2015b	high	Henan	Jan 2010	Dec 2013	Surveillance	ICU	Tertiary	Unclear	√		√
Jia 2011	low	Beijing	Jan 2007	Dec 2009	Surveillance	SICU	Tertiary	Criteria by the Chinese Ministry of Health in 2001		√	√
Jia 2015	moderate	Xinjiang	Jan 2013	Dec 2013	Surveillance	ICU	Tertiary	Criteria by the Chinese Ministry of Health in 2001	√		
Jiang 2012a	low	Jiangsu	May 2010	Oct 2011	Surveillance	General ICU	Tertiary	Criteria by the Chinese Ministry of Health in 2001		√	
Jiang 2012b	moderate	Jiangsu	Jan 2011	Dec 2011	Surveillance	ICU	Tertiary and Non-tertiary	Criteria by the Chinese Ministry of Health in 2001		√	
Jiang 2015	low	Jiangsu	Jan 2010	Dec 2012	Surveillance	General ICU	Tertiary	Criteria by Chinese Thoracic Society in 1999	√	√	√
Kong 2012	moderate	Jiangsu	Jan 2010	Dec 2010	Surveillance	ICU	Non-tertiary	Unclear	√		
Lei 2010	moderate	Jiangxi	Jan 2009	Dec 2009	Surveillance	Neonatal department	Tertiary	Criteria by Chinese Thoracic Society in 1999			√
Li 2011a	moderate	Sichuan	Jan 2009	Dec 2009	Surveillance	NICU	Tertiary	Unclear	√		
Li 2011b	low	Heilongjiang	Jul 2010	Dec 2010	Surveillance	General ICU	Tertiary	Criteria by the Chinese Ministry of Health in 2001	√	√	√
Li 2012	moderate	Sichuan	Jan 2009	Dec 2009	Surveillance	General ICU	Non-tertiary	Unclear	√	√	√
Li 2013a	low	Sichuan	Jan 2010	Jun 2012	Surveillance	General ICU	Tertiary	Criteria by Chinese Thoracic Society in 1999	√		√
Li 2013b	low	Hubei	Jan 2011	Dec 2011	Surveillance	ICU	Tertiary	Criteria by the Chinese Ministry of Health in 2001		√	
Li 2014a	low	Guangxi	Jan 2012	Dec 2013	Surveillance	General ICU	Tertiary	Criteria by the Chinese Ministry of Health in 2001		√	
Li 2014b	low	Guangxi	May 2012	May 2013	Surveillance	General ICU	Tertiary	Criteria by the Chinese Ministry of Health in 2001		√	
Li 2014c	low	Anhui	Jan 2013	Dec 2013	Surveillance	General ICU	Tertiary	Criteria by the Chinese Ministry of Health in 2001	√		√
Li 2015a	low	Sichuan	Jan 2014	Dec 2014	Surveillance	RICU	Tertiary	Criteria by the Chinese Ministry of Health in 2001	√		
Li 2015b	low	Beijing, Shanghai, Chongqing, Zhejiang, Jiangsu,	Oct 2013	Mar 2014	Prospective cohort study	ICU	Tertiary and Non-tertiary	Others		√	

		Henan, Hunan, Guangdong, Shandong, Shanxi, Guizhou, Sichuan, and Liaoning									
Li 2015c	moderate	Jiangsu	Dec 2012	Sep 2014	Prospective cohort study	PICU	Tertiary	Criteria by Chinese Critical Care Medicine Society in 2013	√		√
Li 2015d	moderate	Anhui	Jun 2011	Dec 2012	Surveillance	General ICU	Tertiary	Criteria by the Chinese Ministry of Health in 2001		√	
Liao 2013	low	Guangxi	Jan 2010	Aug 2012	Surveillance	Neonatal department	Tertiary	Criteria by the Chinese Ministry of Health in 2001		√	
Lin 2011	moderate	Henan	Nov 2008	Oct 2009	Surveillance	General ICU	Tertiary	Unclear		√	
Lin 2014	moderate	Henan	Jan 2011	Dec 2011	Surveillance	General ICU	Tertiary	Criteria by the Chinese Ministry of Health in 2001		√	
Lin 2015	high	Guangxi	Jan 2013	Dec 2014	Prospective cohort study	ICU	Tertiary	Unclear	√		√
Liu 2010	low	Inner Mongolia	Jan 2009	Dec 2009	Surveillance	ICU	Tertiary	Criteria by Chinese Thoracic Society in 1999			√
Liu 2011	moderate	Fujian	Apr 2009	Mar 2010	Surveillance	ICU	Tertiary	Criteria by Chinese Thoracic Society in 1999	√		√
Liu 2013a	low	Jiangsu	Jan 2011	Dec 2011	Surveillance	General ICU	Tertiary	Criteria by the Chinese Ministry of Health in 2001		√	
Liu 2013b	low	Shandong	Jan 2012	Dec 2012	Surveillance	General ICU	Non-tertiary	Criteria by the Chinese Ministry of Health in 2001	√		
Liu 2014	low	Hebei	Jan 2012	Dec 2012	Surveillance	General ICU, Neurology ICU, and CCU	Tertiary	Criteria by the Chinese Ministry of Health in 2001	√		√
Liu 2015a	low	Anhui	Jan 2013	Dec 2013	Surveillance	ICU	Tertiary	Criteria by the Chinese Ministry of Health in 2001	√	√	
Liu 2015b	low	Inner Mongolia	Jan 2014	Dec 2014	Surveillance	Unclear	Unclear	Criteria by the Chinese Ministry of Health in 2001	√		
Liu 2015c	low	Shanxi	Jul 2013	Dec 2013	Surveillance	ICU	Tertiary	Criteria by the Chinese	√		

								Ministry of Health in 2001			
Luo 2014	moderate	Fujian	Jan 2012	Dec 2012	Surveillance	Neurosurgery ICU	Tertiary	Criteria by the Chinese Ministry of Health in 2001			√
Ma 2012a	low	Shanxi	Feb 2009	Nov 2010	Surveillance	ICU	Tertiary	Criteria by the Chinese Ministry of Health in 2001			√
Ma 2012b	high	Jiangsu	Jan 2011	Jun 2012	Surveillance	ICU	Tertiary	Unclear		√	
Mai 2013	low	Guangdong	Jan 2012	Sep 2012	Surveillance	Neonatal department	Tertiary	Criteria by the Chinese Ministry of Health in 2001	√		√
Mao 2010	low	Sichuan	Jul 2008	Jun 2009	Surveillance	ICU	Tertiary	Criteria by Chinese Thoracic Society in 1999			√
Mao 2012	low	Shanghai	Jan 2009	Dec 2010	Surveillance	Medical ICU, SICU, Neurosurgery ICU, Cardiac surgery ICU, CCU, PICU, General ICU, Trauma ICU, and Transplantation ICU	Tertiary and Non-tertiary	Criteria by the Chinese Ministry of Health in 2001	√		
Mi 2011	low	Shanghai	Jan 2005	Dec 2009	Surveillance	SICU	Tertiary	Criteria by the Chinese Ministry of Health in 2001		√	
Ming 2012	low	Sichuan	Jan 2011	Jun 2011	Surveillance	ICU	Tertiary	Criteria by the Chinese Ministry of Health in 2001	√		
Pan 2013a	low	Guangdong	Jan 2010	Dec 2012	Surveillance	NICU	Non-tertiary	Criteria by Chinese Thoracic Society in 1999	√		√
Pan 2013b	low	Jiangsu	Jan 2011	Dec 2012	Surveillance	ICU	Tertiary	Criteria by the Chinese Ministry of Health in 2001		√	
Peng 2013	moderate	Sichuan	Feb 2010	Sep 2012	Surveillance	ICU	Tertiary	Others		√	
Pu 2011	moderate	Sichuan	Sep 2010	Feb 2011	Surveillance	General ICU	Tertiary	Criteria by the Chinese Ministry of Health in 2001		√	
Qi 2010a	low	Zhejiang	Mar 2008	Sep 2009	Surveillance	EICU	Non-tertiary	Criteria by Chinese Thoracic Society in 1999			√
Qi 2010b	moderate	Hebei	Jun 2008	Oct 2009	Prospective cohort study	RICU	Tertiary	Others			√
Qi 2013	low	Anhui	Jan 2011	Dec 2011	Surveillance	General ICU	Tertiary	Criteria by the Chinese Ministry of Health in 2001			√
Qian 2015	moderate	Anhui	Jan 2011	Dec 2011	Surveillance	General ICU	Tertiary	Unclear	√		√
Qu 2011	low	Henan	Dec 2009	Nov 2010	Surveillance	Neurosurgery	Tertiary	Criteria by the Chinese	√		

						emergency room		Ministry of Health in 2001			
Qu 2015	moderate	Beijing	Sep 2010	Aug 2012	Prospective cohort study	PICU	Tertiary	Criteria by Chinese Critical Care Medicine Society in 2013			√
Ren 2012	low	Beijing	Feb 2010	Sep 2011	Surveillance	NICU	Tertiary	Criteria by the Chinese Ministry of Health in 2001	√		
Ren 2015	low	Beijing, Shandong, Shanxi,Chongqing,Zhejiang, Jiangsu, Henan, Hunan, and Guangdong	Oct 2013	Sep 2014	Surveillance	NICU	Tertiary	Criteria by the Chinese Ministry of Health in 2001	√		
Shen 2010	low	Jiangsu	Nov 2008	Apr 2009	Surveillance	General ICU	Tertiary	Criteria by the Chinese Ministry of Health in 2001		√	
Sheng 2015	low	Anhui	Jan 2013	Dec 2013	Surveillance	General ICU	Tertiary	Criteria by the Chinese Ministry of Health in 2001	√		√
Shu 2015a	moderate	Anhui	Apr 2012	Apr 2013	Prospective cohort study	Unclear	Tertiary	Others	√		√
Shu 2015b	low	Anhui	Oct 2012	Sep 2013	Prospective cohort study	NICU	Tertiary	Others	√		
Situ 2015	moderate	Guangdong	Jan 2011	Dec 2013	Surveillance	NICU	Tertiary	Criteria by Chinese Thoracic Society in 1999			√
Song 2013	low	Jiangsu	Sep 2010	Sep 2011	Surveillance	ICU	Tertiary	Criteria by the Chinese Ministry of Health in 2001	√		√
Song 2015	low	Hebei	Jan 2012	Dec 2012	Surveillance	ICU	Tertiary	Criteria by the Chinese Ministry of Health in 2001	√		
Sun 2015	low	Sichuan	Apr 2013	Jul 2013	Surveillance	ICU	Tertiary	Others	√		
Tang 2012a	moderate	Sichuan	May 2011	Oct 2011	Surveillance	ICU	Non-tertiary	Unclear	√	√	
Tang 2012b	low	Jiangxi	Jun 2006	May 2007	Surveillance	ICU	Tertiary	Criteria by the Chinese Ministry of Health in 2001			√
Tang 2013	low	Jiangsu	Jan 2010	Dec 2011	Surveillance	NICU	Tertiary	Criteria by the Chinese Ministry of Health in 2001			√
Tang 2015	moderate	Jiangsu	May 2014	Sep 2014	Surveillance	ICU	Non-tertiary	Unclear	√		
Wang 2010a	high	Beijing	Oct 2009	Dec 2009	Prospective	Unclear	Tertiary	Unclear			√

					cohort study						
Wang 2010b	moderate	Guangdong	Dec 2006	Oct 2007	Surveillance	General ICU	Tertiary	Criteria by Chinese Thoracic Society in 1999			√
Wang 2010c	moderate	Shanghai	Jul 2006	Jun 2009	Surveillance	ICU	Tertiary	Criteria by Chinese Thoracic Society in 1999			√
Wang 2011a	moderate	Xinjiang	Jan 2009	Dec 2009	Surveillance	SICU	Tertiary	Criteria by the Chinese Ministry of Health in 2001	√		
Wang 2011b	moderate	Shaanxi	Sep 2010	Dec 2010	Surveillance	Emergency room and ICU	Tertiary	Criteria by Chinese Thoracic Society in 1999	√		√
Wang 2011c	moderate	Hebei	May 2010	Mar 2011	Prospective cohort study	RICU	Tertiary	Criteria by the Chinese Ministry of Health in 2001			√
Wang 2012	moderate	Henan	Jan 2009	Dec 2010	Surveillance	Cardiac surgical ICU	Tertiary	Criteria by Chinese Thoracic Society in 1999			√
Wang 2013a	low	Jiangsu	Jan 2012	Dec 2012	Surveillance	NICU	Non-tertiary	Criteria by the Chinese Ministry of Health in 2001	√		
Wang 2013b	moderate	Unclear	Jan 2011	Dec 2011	Surveillance	ICU	Tertiary	Criteria by the Chinese Ministry of Health in 2001		√	
Wang 2013c	moderate	Shandong	Oct 2009	Jan 2013	Surveillance	ICU	Non-tertiary	Criteria by Chinese Thoracic Society in 1999			√
Wang 2013d	moderate	Henan	Jan 2012	Dec 2012	Surveillance	General ICU	Tertiary	Criteria by the Chinese Ministry of Health in 2001		√	
Wang 2014a	moderate	Henan	Jul 2011	Jun 2013	Surveillance	General ICU	Tertiary	Criteria by Chinese Thoracic Society in 1999	√		√
Wang 2014b	moderate	Jiangsu	Jan 2012	Dec 2012	Surveillance	ICU	Tertiary	Criteria by the Chinese Ministry of Health in 2001		√	√
Wang 2014c	low	Zhejiang	Jan 2011	Dec 2012	Surveillance	Neonatal department	Tertiary	Criteria by the Chinese Ministry of Health in 2001	√		√
Wang 2015a	moderate	Zhejiang	Jan 2012	Sep 2013	Surveillance	General ICU	Tertiary	Criteria by the Chinese Ministry of Health in 2001	√		
Wang 2015b	low	Zhejiang	Feb 2012	Feb 2013	Surveillance	ICU	Tertiary	Criteria by the Chinese Ministry of Health in 2001		√	
Wang 2015c	low	Hubei	Mar 2012	Mar 2013	Surveillance	NICU	Tertiary	Criteria by the Chinese Ministry of Health in 2001	√		
Wang 2015d	low	Xinjiang	Jan 2011	Dec 2013	Surveillance	NICU	Tertiary	Others	√		√
Wei 2010	moderate	Guangdong	Jun 2008	Nov 2008	Surveillance	NICU	Tertiary	Criteria by the Chinese Ministry of Health in 2001		√	

Wei 2012	moderate	Xinjiang	Mar 2011	Aug 2011	Surveillance	ICU	Tertiary	Criteria by the Chinese Ministry of Health in 2001		√	
Wei 2013	moderate	Xinjiang	Mar 2012	Aug 2012	Surveillance	ICU	Tertiary	Criteria by the Chinese Ministry of Health in 2001	√		
Wen 2013	moderate	Anhui	Jun 2010	Jun 2011	Prospective cohort study	ICU	Non-tertiary	Criteria by Chinese Thoracic Society in 1999			√
Wu 2012	low	Guangdong	Jan 2010	Sep 2010	Surveillance	NICU	Non-tertiary	Criteria by the Chinese Ministry of Health in 2001	√		√
Wu 2013	low	Zhejiang	Jan 2009	Dec 2012	Surveillance	ICU	Tertiary	Criteria by Chinese Thoracic Society in 1999	√		
Wu 2015	low	Hebei	Sep 2013	Aug 2014	Surveillance	General ICU	Tertiary	Criteria by the Chinese Ministry of Health in 2001	√		
Xiang 2014	moderate	Sichuan	Jan 2012	Dec 2012	Surveillance	ICU	Tertiary	Criteria by the Chinese Ministry of Health in 2001		√	
Xie 2011	high	Jiangsu	Nov 2009	Feb 2010	Surveillance	ICU	Unclear	Unclear			√
Xie 2014	moderate	Hubei	Jan 2007	Dec 2010	Surveillance	ICU	Tertiary	Unclear	√		√
Xing 2012	low	Anhui	Apr 2010	Sep 2010	Surveillance	ICU	Non-tertiary	Criteria by the Chinese Ministry of Health in 2001	√		
Xing 2013	low	Anhui	Feb 2012	Jul 2012	Surveillance	NICU	Non-tertiary	Criteria by Chinese Thoracic Society in 1999	√		√
Xu 2010a	low	Hebei	Jan 2006	Dec 2008	Surveillance	ICU	Tertiary	Criteria by Chinese Thoracic Society in 1999			√
Xu 2010b	low	Jiangxi	Feb 2009	Jan 2010	Surveillance	Neonatal department	Tertiary	Criteria by the Chinese Ministry of Health in 2001		√	
Xu 2014a	moderate	Xinjiang	Jan 2012	Dec 2012	Surveillance	ICU	Non-tertiary	Criteria by the Chinese Ministry of Health in 2001	√		
Xu 2014b	moderate	Hubei	Jan 2011	Dec 2012	Surveillance	PICU	Tertiary	Others			√
Xue 2014	moderate	Hubei	Oct 2010	Sep 2012	Surveillance	General ICU	Tertiary	Criteria by the Chinese Ministry of Health in 2001	√		
Xue 2015	moderate	Shanxi	Jan 2009	Dec 2012	Prospective cohort study	ICU	Non-tertiary	Criteria by Chinese Thoracic Society in 1999			√
Yan 2014	moderate	Unclear	Jan 2011	Dec 2012	Surveillance	ICU	Unclear	Criteria by the Chinese Ministry of Health in 2001		√	
Yang 2012	low	Zhejiang	Jan 2010	Dec 2010	Surveillance	ICU	Tertiary	Criteria by Chinese Thoracic Society in 1999	√		√
Yang 2013	low	Jiangsu	Jan 2011	Dec 2011	Surveillance	General ICU	Non-tertiary	Criteria by the Chinese	√		

								Ministry of Health in 2001			
Yang 2014a	moderate	Beijing	Jun 2010	Dec 2010	Surveillance	ICU	Tertiary	Criteria by Chinese Thoracic Society in 1999			√
Yang 2014b	high	Gansu	Jan 2013	Dec 2013	Prospective cohort study	ICU	Tertiary	Unclear			√
Yang 2014c	moderate	Beijing	Jun 2007	May 2010	Prospective cohort study	ICU	Tertiary	Others	√		√
Yang 2014d	low	Jiangxi	Jan 2012	Aug 2013	Surveillance	General ICU	Tertiary	Criteria by the Chinese Ministry of Health in 2001		√	
Yang 2015a	moderate	Hebei	Jan 2012	Dec 2013	Surveillance	ICU	Tertiary	Criteria by the Chinese Ministry of Health in 2001	√		
Yang 2015b	moderate	Jiangsu	Jun 2013	May 2014	Prospective cohort study	ICU	Tertiary	Criteria by Chinese Critical Care Medicine Society in 2013	√		√
Yao 2015	moderate	Hebei	Jan 2014	Dec 2014	Surveillance	ICU	Tertiary	Criteria by the Chinese Ministry of Health in 2001	√		
Ye 2014	moderate	Zhejiang	Jan 2010	Sep 2013	Surveillance	ICU	Tertiary	Criteria by Chinese Thoracic Society in 1999	√		√
Ying 2011	low	Shandong	Jan 2008	Dec 2010	Surveillance	NICU	Tertiary	Criteria by the Chinese Ministry of Health in 2001		√	
Ying 2013	moderate	Shandong	Jan 2008	Dec 2008	Surveillance	NICU	Tertiary	Criteria by the Chinese Ministry of Health in 2001		√	
You 2012	moderate	Jiangxi	Sep 2009	Mar 2011	Surveillance	ICU	Non-tertiary	Criteria by Chinese Thoracic Society in 1999			√
Yu 2011a	low	Henan	May 2008	Jan 2010	Surveillance	RICU	Tertiary	Criteria by Chinese Thoracic Society in 1999			√
Yu 2011b	low	Zhejiang	Jan 2010	Dec 2010	Surveillance	ICU	Tertiary	Criteria by the Chinese Ministry of Health in 2001			√
Yu 2012a	low	Shanghai	Jan 2008	Dec 2011	Surveillance	ICU	Tertiary	Criteria by Chinese Thoracic Society in 1999			√
Yu 2012b	moderate	Anhui	Jan 2009	Dec 2009	Surveillance	ICU	Tertiary	Criteria by the Chinese Ministry of Health in 2001		√	
Yu 2014	high	Jiangxi	Apr 2010	Apr 2012	Surveillance	ICU	Non-tertiary	Unclear	√		√
Yu 2015a	moderate	Jiangxi	Aug 2012	Jul 2013	Surveillance	ICU	Tertiary	Criteria by the Chinese Ministry of Health in 2001	√		√
Yu 2015b	low	Shanghai	Jun 2009	Jun 2012	Prospective	ICU	Tertiary	Criteria by Chinese	√		√

					cohort study			Thoracic Society in 1999			
Yu 2015c	moderate	Jiangsu	Jan 2011	Dec 2014	Surveillance	General ICU and SICU	Tertiary	Criteria by Chinese Thoracic Society in 1999	√	√	√
Yuan 2011	low	Beijing	Jan 2008	Dec 2010	Surveillance	SICU	Tertiary	Criteria by the Chinese Ministry of Health in 2001	√		
Yue 2015	moderate	Shandong	Jan 2010	Aug 2012	Prospective cohort study	ICU	Tertiary and Non-tertiary	Unclear	√		
Zeng 2014	low	Sichuan	Apr 2013	Jul 2013	Surveillance	ICU	Tertiary	Others			√
Zhang 2010a	low	Shanghai	Sep 2004	Dec 2009	Surveillance	SICU, Cardiac surgery ICU, CCU, Medical ICU, EICU and RICU	Tertiary	Criteria by the Chinese Ministry of Health in 2001	√		√
Zhang 2010b	high	Guangxi	Jan 2009	Dec 2009	Surveillance	Neonatal department	Tertiary	Unclear			√
Zhang 2011a	low	Zhejiang	Feb 2009	Jan 2010	Surveillance	ICU	Tertiary	Criteria by the Chinese Ministry of Health in 2001		√	
Zhang 2011b	moderate	Shanxi	Nov 2008	Nov 2009	Surveillance	ICU	Tertiary	Criteria by the Chinese Ministry of Health in 2001	√		√
Zhang 2011c	low	Chongqing	Jan 2010	Dec 2010	Surveillance	ICU	Tertiary	Criteria by the Chinese Ministry of Health in 2001	√		
Zhang 2011d	low	Chongqing	Jan 2010	Dec 2010	Surveillance	RICU	Tertiary	Criteria by the Chinese Ministry of Health in 2001	√		
Zhang 2011e	low	Guangxi	Jan 2009	Dec 2010	Surveillance	ICU	Tertiary	Criteria by the Chinese Ministry of Health in 2001		√	
Zhang 2011f	moderate	Fujian	Apr 2008	Dec 2008	Surveillance	SICU	Tertiary	Criteria by Chinese Thoracic Society in 1999	√		
Zhang 2012a	moderate	Shandong	Jul 2010	Mar 2012	Prospective cohort study	ICU	Tertiary	Criteria by Chinese Thoracic Society in 1999			√
Zhang 2012b	moderate	Fujian	Jul 2010	Jun 2011	Surveillance	ICU	Tertiary	Criteria by the Chinese Ministry of Health in 2001	√		
Zhang 2013a	moderate	Jiangxi	Jan 2010	Jan 2013	Surveillance	ICU	Tertiary	Criteria by Chinese Thoracic Society in 1999			√
Zhang 2013b	low	Shaanxi	Jul 2011	Jun 2012	Surveillance	ICU	Non-tertiary	Criteria by the Chinese Ministry of Health in 2001	√		
Zhang 2014a	low	Henan	Jan 2012	Dec 2012	Surveillance	ICU	Tertiary	Criteria by the Chinese Ministry of Health in 2001	√		
Zhang 2014b	moderate	Henan	Jan 2014	Jun 2014	Surveillance	NICU	Tertiary	Criteria by the Chinese	√		

								Ministry of Health in 2001			
Zhang 2014c	low	Jiangsu	Jan 2011	Dec 2011	Surveillance	NICU	Tertiary	Criteria by the Chinese Ministry of Health in 2001	√		
Zhang 2015a	moderate	Hebei	Jan 2013	Jan 2014	Prospective cohort study	ICU	Tertiary	Criteria by Chinese Thoracic Society in 1999			√
Zhang 2015b	high	Xinjiang	Jan 2010	Dec 2014	Surveillance	ICU	Tertiary	Unclear	√		
Zhang 2015c	moderate	Chognqing	Jan 2012	Sep 2012	Prospective cohort study	PICU	Tertiary	Criteria by Chinese Thoracic Society in 1999	√		√
Zhao 2012a	moderate	Guangdong	Jan 2009	Dec 2011	Surveillance	Unclear	Tertiary	Others			√
Zhao 2012b	moderate	Hebei	Jan 2012	Jul 2012	Surveillance	ICU	Tertiary	Criteria by the Chinese Ministry of Health in 2001		√	
Zhao 2013	low	Hubei	Jan 2012	Dec 2012	Surveillance	ICU	Tertiary	Criteria by the Chinese Ministry of Health in 2001	√		
Zhao 2014a	low	Guangdong	Jan 2012	Jan 2012	Surveillance	ICU	Tertiary	Others			√
Zhao 2014b	moderate	Guangdong	Jan 2012	Dec 2012	Surveillance	Pediatric cardiac ICU	Tertiary	Unclear	√		
Zhao 2015	moderate	Hebei	Jan 2013	Dec 2013	Prospective cohort study	ICU	Tertiary	Criteria by the Chinese Ministry of Health in 2001	√		
Zhong 2013	high	Sichuan	Jan 2011	Oct 2011	Prospective cohort study	ICU	Non-tertiary	Unclear			√
Zhou 2013a	moderate	Jiangsu	Jan 2011	Dec 2012	Surveillance	ICU	Tertiary and Non-tertiary	Criteria by the Chinese Ministry of Health in 2001		√	
Zhou 2013b	high	Chongqing	Jan 2011	Dec 2011	Surveillance	ICU	Non-tertiary	Unclear	√		
Zhou 2013c	moderate	Guizhou	Jan 2011	Dec 2012	Surveillance	ICU	Tertiary	Criteria by the Chinese Ministry of Health in 2001		√	
Zhu 2010	moderate	Ningxia	Apr 2008	Mar 2009	Surveillance	Neurology ICU	Tertiary	Criteria by the Chinese Ministry of Health in 2001	√		
Zhu 2014a	moderate	Tianjin	Jan 2012	Dec 2012	Surveillance	ICU	Tertiary	Criteria by the Chinese Ministry of Health in 2001	√		
Zhu 2014b	moderate	Xinjiang	Jan 2010	Dec 2012	Surveillance	ICU	Tertiary	Unclear	√		√
Zong 2013	low	Jiangsu	Jul 2010	Jun 2011	Surveillance	ICU	Tertiary	Criteria by the Chinese Ministry of Health in 2001		√	
Zou 2013	moderate	Hubei	Jan 2011	Dec 2012	Surveillance	ICU	Tertiary	Criteria by the Chinese Ministry of Health in 2001	√		
Zou 2015	low	Guangdong	May 2014	May 2015	Surveillance	PICU	Tertiary	Criteria by the Chinese Ministry of Health in 2001	√		

Zuo 2015	low	Guangdong	Jan 2011	Dec 2013	Surveillance	NICU	Tertiary	Criteria by Chinese Thoracic Society in 1999	√

[a]ICU=intensive care unit, NICU=neonatal intensive care unit, PICU=pediatric intensive care unit, RICU=respiratory intensive care unit, SICU= surgery intensive care unit, CCU=cardiac care unit, EICU=emergency intensive care unit.

[b]Others refer to a specified diagnostic criteria rather than the one proposed by Chinese Thoracic Society, Chinese Critical Care Medicine Society, or the Chinese Ministry of Health, which was reported in a small number of studies.

Table S4. The results of subgroup analyses by Chinese provinces and years of study

Subgroups		Studies	Sample size	Estimate (%)[a]	95%CI (%)	I^2 (%)	*p* value[b]
Overall[c]		93	71936	23.8	20.6-27.2	98.9	-
Province	Anhui	7	1611	22.5	9.8-38.5	97.5	<0.001
	Beijing	5	370	28.1	23.6-32.7	0.0	
	Chongqing	1	30	70.0	52.7-84.8	-	
	Fujian	3	1201	7.5	5.9-9.4	22.4	
	Gansu	1	54	29.6	18.3-42.4	-	
	Guangdong	8	3931	16.9	10.9-24.0	96.0	
	Guangxi	3	340	31.2	12.3-54.1	91.6	
	Guizhou	1	319	25.7	21.1-30.6	-	
	Hebei	6	1206	27.8	13.4-45.0	96.6	
	Heilongjiang	2	82	20.7	12.7-30.1	0.0	
	Henan	4	2083	17.7	11.3-25.2	92.1	
	Hubei	4	4760	24.7	14.4-36.8	96.1	
	Jiangsu	8	8464	18.8	10.4-28.9	98.9	
	Jiangxi	10	11185	29.5	17.0-43.7	97.3	
	Inner Mongolia	1	213	32.9	26.7-39.3	-	
	Qinghai	1	146	14.4	9.2-20.5	-	
	Shaanxi	1	96	16.7	9.9-24.7	-	
	Shandong	3	1128	27.5	8.8-51.7	97.8	
	Shanghai	4	29715	17.8	5.4-35.2	99.2	
	Shanxi	4	559	39.8	30.5-49.5	81.2	
	Sichuan	7	2227	15.0	8.3-23.4	95.4	
	Xinjiang	2	819	34.5	28.4-40.9	69.1	
	Zhejiang	7	1277	30.3	22.1-39.3	90.2	
Year of study	2006	1	105	49.5	40.0-59.0	-	<0.001
	2007	5	29436	33.8	7.8-67.0	99.6	
	2008	7	5213	29.2	21.1-38.1	94.7	
	2009	19	6672	22.2	17.7-27.0	94.0	
	2010	22	12805	21.4	15.7-27.7	98.2	
	2011	22	7170	24.6	18.0-32.0	97.6	
	2012	17	4848	16.0	11.0-21.8	96.0	
	2013	12	1734	19.3	14.0-25.2	87.7	
	2014	1	56	19.6	10.4-31.0	-	

CI, confidence interval.

[a]Pooled estimates were calculated by a random-effects model.

[b]The Q test for heterogeneity was used to compare the incidence across subgroups.

[c] Studies that reported the cumulative incidence of VAP

Table S5. Summary of diagnostic requirements for the three published sets of criteria in mainland China

Published criteria	Clinical and radiography criteria	Microbiology criteria
the Chinese Ministry of Health in 2001	• Cough, sputum or moist rales<br>• At least one criterion:<br>− Fever<br>− Elevated white blood cell count and/or neutrophil percentage<br>− Inflammatory infiltrates on chest X-ray	• At least one criterion:<br>− The same pathogens detected in two consecutive sputum samples<br>− Quantitative sputum culture ≥ 10^6 cfu/ml<br>− Positive growth in blood culture or culture of pleural fluid<br>− Lower respiratory tract secretions collected via bronchoscopy be cultured for microorganisms ≥ 10^5 cfu/ml, BAL samples be cultured for microorganisms ≥ 10^4 cfu/ml, or pathogens detected in PSB or PBAL samples<br>− Uncommon pathogens detected in sputum or lower respiratory tract samples<br>− Abnormal immunology tests or histopathological evidence of pneumonia
Chinese Thoracic Society in 1999	• At least one criterion:<br>− The advent of cough, sputum or existing respiratory symptoms, and purulent sputum<br>− Fever<br>− Pulmonary consolidation syndrome and/or moist rales<br>− White blood cell count > 10×10^9/ L or < 4×10^9/L, with or without an accompanying shift to the left<br>• Patchy infiltrate shadows or interstitial change on chest X-ray, with or without pleural effusion	—
Chinese Critical Care Medicine Society in 2013	• New or progressive infiltrates on chest X-ray<br>• At least two criteria:<br>− Temperature > 38℃ or < 36℃<br>− White blood cell count > 10×10^9/ L or < 4×10^9/L<br>− Purulent secretions	—

Study	Events	Total	Events	95%-CI	Weight
Chen 2010b	82	319	25.7	[21.0; 30.9]	1.1%
Chen 2010c	33	132	25.0	[17.9; 33.3]	1.1%
Chen 2011a	48	106	45.3	[35.6; 55.2]	1.1%
Chen 2012	69	125	55.2	[46.0; 64.1]	1.1%
Chen 2013a	20	60	33.3	[21.7; 46.7]	1.0%
Chen 2013b	1041	10402	10.0	[9.4; 10.6]	1.2%
Chen 2013d	89	727	12.2	[9.9; 14.8]	1.1%
Chen 2014	48	106	45.3	[35.6; 55.2]	1.1%
Deng 2015	96	846	11.3	[9.3; 13.7]	1.1%
Ding 2011	21	146	14.4	[9.1; 21.1]	1.1%
Feng 2013	89	349	25.5	[21.0; 30.4]	1.1%
Gao 2011	62	173	35.8	[28.7; 43.5]	1.1%
Gao 2015a	11	56	19.6	[10.2; 32.4]	1.0%
He 2015	13	45	28.9	[16.4; 44.3]	1.0%
Hu 2012	48	300	16.0	[12.0; 20.6]	1.1%
Huang 2011a	24	153	15.7	[10.3; 22.4]	1.1%
Huang 2011b	48	694	6.9	[5.1; 9.1]	1.1%
Huang 2012	22	215	10.2	[6.5; 15.1]	1.1%
Huang 2013	40	253	15.8	[11.5; 20.9]	1.1%
Huang 2015b	97	899	10.8	[8.8; 13.0]	1.1%
Jia 2011	25	85	29.4	[20.0; 40.3]	1.1%
Jiang 2015	112	2231	5.0	[4.2; 6.0]	1.2%
Lei 2010	7	20	35.0	[15.4; 59.2]	0.8%
Li 2011b	6	26	23.1	[9.0; 43.6]	0.9%
Li 2012	17	96	17.7	[10.7; 26.8]	1.1%
Li 2013a	201	756	26.6	[23.5; 29.9]	1.1%
Li 2014c	36	207	17.4	[12.5; 23.3]	1.1%
Li 2015c	27	66	40.9	[29.0; 53.7]	1.0%
Lin 2015	14	42	33.3	[19.6; 49.5]	1.0%
Liu 2010	70	213	32.9	[26.6; 39.6]	1.1%
Liu 2011	20	301	6.6	[4.1; 10.1]	1.1%
Liu 2014	22	346	6.4	[4.0; 9.5]	1.1%
Luo 2014	48	685	7.0	[5.2; 9.2]	1.1%
Ma 2012a	72	141	51.1	[42.5; 59.6]	1.1%
Mai 2013	4	42	9.5	[2.7; 22.6]	1.0%
Mao 2010	78	419	18.6	[15.0; 22.7]	1.1%
Pan 2013a	28	139	20.1	[13.8; 27.8]	1.1%
Qi 2010a	96	171	56.1	[48.4; 63.7]	1.1%
Qi 2010b	16	50	32.0	[19.5; 46.7]	1.0%
Qi 2013	60	168	35.7	[28.5; 43.5]	1.1%
Qian 2015	24	827	2.9	[1.9; 4.3]	1.1%
Qu 2015	19	60	31.7	[20.3; 45.0]	1.0%
Sheng 2015	39	261	14.9	[10.8; 19.9]	1.1%
Shu 2015a	18	35	51.4	[34.0; 68.6]	1.0%
Situ 2015	198	816	24.3	[21.4; 27.4]	1.1%
Song 2013	86	178	48.3	[40.8; 55.9]	1.1%
Tang 2012b	52	105	49.5	[39.6; 59.5]	1.1%
Tang 2013	8	2095	0.4	[0.2; 0.8]	1.2%
Wang 2010a	10	38	26.3	[13.4; 43.1]	1.0%
Wang 2010b	16	25	64.0	[42.5; 82.0]	0.9%
Wang 2010c	48	121	39.7	[30.9; 49.0]	1.1%
Wang 2011b	16	96	16.7	[9.8; 25.6]	1.1%
Wang 2011c	15	47	31.9	[19.1; 47.1]	1.0%
Wang 2012	180	1015	17.7	[15.4; 20.2]	1.2%
Wang 2013c	59	126	46.8	[37.9; 55.9]	1.1%
Wang 2014a	9	64	14.1	[6.6; 25.0]	1.0%
Wang 2014b	36	204	17.6	[12.7; 23.6]	1.1%
Wang 2014c	5	29	17.2	[5.8; 35.8]	0.9%
Wang 2015d	211	564	37.4	[33.4; 41.6]	1.1%
Wen 2013	32	71	45.1	[33.2; 57.3]	1.1%
Wu 2012	25	136	18.4	[12.3; 25.9]	1.1%
Xie 2011	2	7	28.6	[3.7; 71.0]	0.6%
Xie 2014	868	4155	20.9	[19.7; 22.2]	1.2%
Xing 2013	4	42	9.5	[2.7; 22.6]	1.0%
Xu 2010a	237	651	36.4	[32.7; 40.2]	1.1%
Xu 2014b	24	180	13.3	[8.7; 19.2]	1.1%
Xue 2015	33	75	44.0	[32.5; 55.9]	1.1%
Yang 2012	45	141	31.9	[24.3; 40.3]	1.1%
Yang 2014a	32	109	29.4	[21.0; 38.8]	1.1%
Yang 2014b	16	54	29.6	[18.0; 43.6]	1.0%
Yang 2014c	18	78	23.1	[14.3; 34.0]	1.1%
Yang 2015b	21	48	43.8	[29.5; 58.8]	1.0%
Ye 2014	87	351	24.8	[20.4; 29.6]	1.1%
You 2012	4	40	10.0	[2.8; 23.7]	1.0%
Yu 2011a	34	105	32.4	[23.6; 42.2]	1.1%
Yu 2011b	51	191	26.7	[20.6; 33.6]	1.1%
Yu 2012a	112	1001	11.2	[9.3; 13.3]	1.2%
Yu 2014	16	96	16.7	[9.8; 25.6]	1.1%
Yu 2015a	18	72	25.0	[15.5; 36.6]	1.1%
Yu 2015b	20	60	33.3	[21.7; 46.7]	1.0%
Yu 2015c	265	3635	7.3	[6.5; 8.2]	1.2%
Zeng 2014	1	49	2.0	[0.1; 10.9]	1.0%
Zhang 2010a	481	28533	1.7	[1.5; 1.8]	1.2%
Zhang 2010b	22	45	48.9	[33.7; 64.2]	1.0%
Zhang 2011b	51	170	30.0	[23.2; 37.5]	1.1%
Zhang 2012a	45	156	28.8	[21.9; 36.6]	1.1%
Zhang 2013a	48	106	45.3	[35.6; 55.2]	1.1%
Zhang 2015a	18	52	34.6	[22.0; 49.1]	1.0%
Zhang 2015c	21	30	70.0	[50.6; 85.3]	0.9%
Zhao 2012a	113	1317	8.6	[7.1; 10.2]	1.2%
Zhao 2014a	45	729	6.2	[4.5; 8.2]	1.1%
Zhong 2013	15	60	25.0	[14.7; 37.9]	1.0%
Zhu 2014b	79	255	31.0	[25.4; 37.0]	1.1%
**Random effects model**		**71816**	**23.8**	**[20.6; 27.2]**	**100.0%**

Heterogeneity: I^2 = 98.9%, p < 0.0001

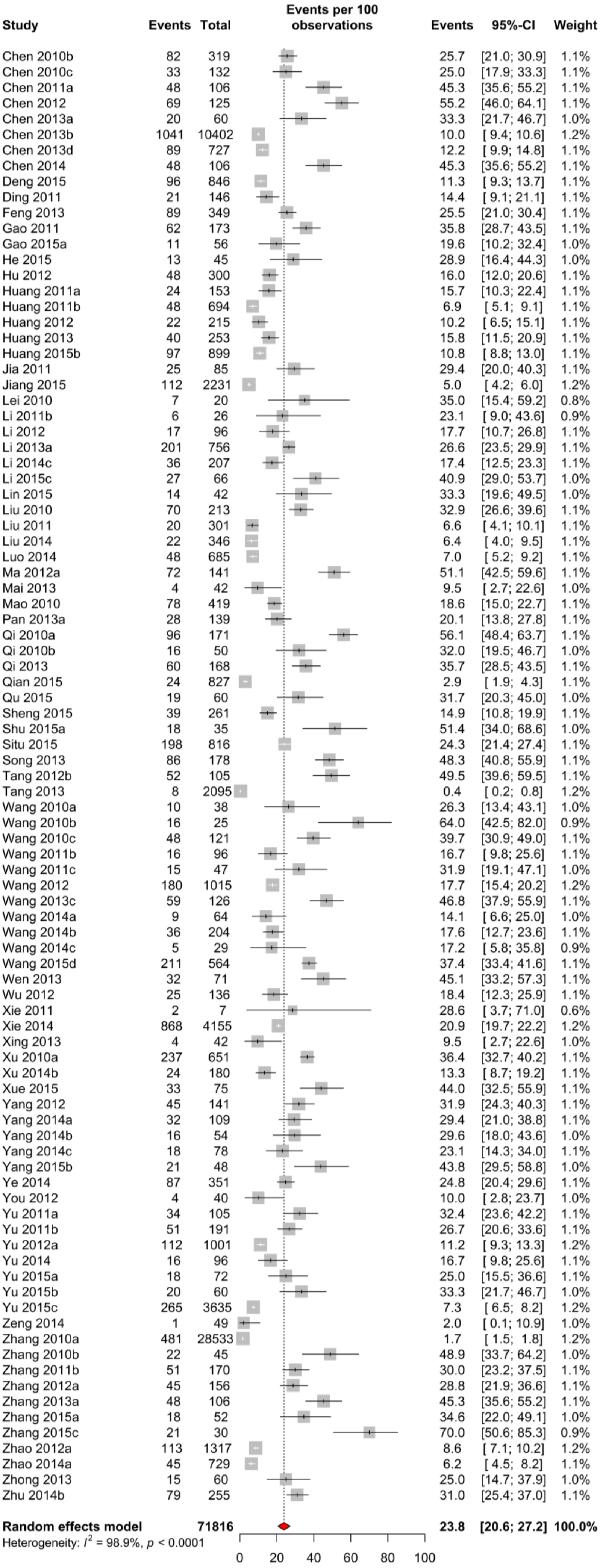

Figure S1. Forest plot of the cumulative incidence of ventilator-associated pneumonia (VAP) using a random-effects model.

Study	VAPs	Ventilator-days	VAPs per 1000 ventilator-days	95%CI	W(random)
Cai 2010	38	778	48.84	[34.79; 66.43]	2.1%
Chen 2010a	17	646	26.32	[15.40; 41.80]	1.8%
Chen 2011b	121	2393	50.56	[42.13; 60.12]	2.3%
Chen 2013c	5	73	68.49	[22.61; 152.65]	1.2%
Chen 2015a	3	358	8.38	[1.73; 24.29]	0.9%
Chen 2015b	24	1862	12.89	[8.28; 19.12]	2.0%
Gao 2014	134	3851	34.80	[29.23; 41.08]	2.3%
Jia 2011	25	920	27.17	[17.66; 39.85]	2.0%
Jiang 2012a	56	2731	20.51	[15.53; 26.55]	2.2%
Jiang 2012b	3089	162667	18.99	[18.33; 19.66]	2.4%
Jiang 2015	113	6071	18.61	[15.36; 22.34]	2.3%
Li 2011b	7	288	24.31	[9.83; 49.44]	1.4%
Li 2012	19	182	104.40	[64.03; 158.22]	1.9%
Li 2013b	38	1581	24.04	[17.06; 32.84]	2.1%
Li 2014a	46	1416	32.49	[23.88; 43.10]	2.2%
Li 2014b	38	1589	23.91	[16.98; 32.68]	2.1%
Li 2015b	372	39057	9.52	[8.59; 10.54]	2.4%
Li 2015d	156	4135	37.73	[32.13; 43.99]	2.3%
Liao 2013	31	2368	13.09	[8.91; 18.53]	2.1%
Lin 2011	53	2447	21.66	[16.27; 28.24]	2.2%
Lin 2014	67	2861	23.42	[18.19; 29.65]	2.2%
Liu 2013a	42	2258	18.60	[13.44; 25.06]	2.1%
Liu 2015a	35	1619	21.62	[15.10; 29.94]	2.1%
Ma 2012b	16	933	17.15	[9.83; 27.70]	1.8%
Mi 2011	136	8771	15.51	[13.02; 18.32]	2.3%
Pan 2013b	15	3049	4.92	[2.76; 8.10]	1.8%
Peng 2013	4	38	105.26	[29.43; 248.05]	1.1%
Pu 2011	138	5016	27.51	[23.16; 32.42]	2.3%
Shen 2010	30	429	69.93	[47.68; 98.33]	2.1%
Tang 2012a	1	7	142.86	[3.61; 578.72]	0.4%
Wang 2013b	34	690	49.28	[34.36; 68.18]	2.1%
Wang 2013d	34	1661	20.47	[14.22; 28.49]	2.1%
Wang 2014b	37	2058	17.98	[12.69; 24.70]	2.1%
Wang 2015b	220	2805	78.43	[68.75; 89.01]	2.4%
Wei 2010	37	8776	4.22	[2.97; 5.81]	2.1%
Wei 2012	274	5722	47.89	[42.50; 53.74]	2.4%
Xiang 2014	51	1870	27.27	[20.37; 35.70]	2.2%
Xu 2010b	17	707	24.05	[14.07; 38.22]	1.8%
Yan 2014	35	1735	20.17	[14.09; 27.94]	2.1%
Yang 2014d	8	668	11.98	[5.18; 23.46]	1.4%
Ying 2011	22	1202	18.30	[11.50; 27.58]	1.9%
Ying 2013	11	297	37.04	[18.63; 65.30]	1.6%
Yu 2012b	101	3976	25.40	[20.74; 30.78]	2.3%
Yu 2015c	267	17232	15.49	[13.70; 17.45]	2.4%
Zhang 2011a	46	1398	32.90	[24.19; 43.65]	2.2%
Zhang 2011e	109	3808	28.62	[23.56; 34.43]	2.3%
Zhao 2012b	8	379	21.11	[9.16; 41.17]	1.5%
Zhou 2013a	6290	366002	17.19	[16.77; 17.61]	2.4%
Zhou 2013c	147	6089	24.14	[20.43; 28.31]	2.3%
Zong 2013	12	1391	8.63	[4.47; 15.02]	1.7%
**Random effects model**		**688860**	**24.14**	**[21.19; 27.51]**	**100%**

Heterogeneity: I-squared=97.1%, p<0.0001

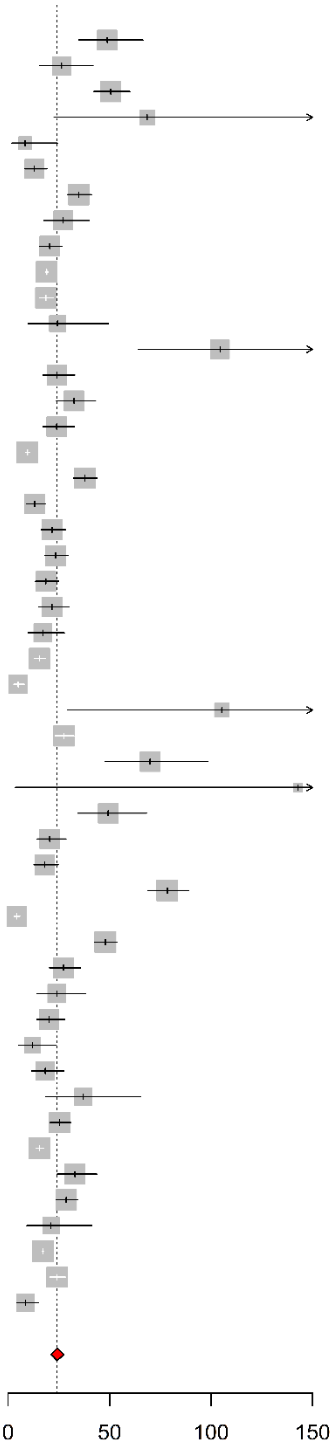

Figure S2. Forest plot of the incidence density (reported as episodes per 1000 ventilator-days) of ventilator-associated pneumonia (VAP) using a random-effects model.

Study	VAP patients	Ventilator-days	VAP patients per 1000 ventilator-days	95%CI	W(random)
Cai 2015	78	327	238.53	[193.36; 288.51]	1.1%
Chen 2012	69	1477	46.72	[36.53; 58.75]	1.1%
Chen 2013b	1041	46095	22.58	[21.25; 23.98]	1.1%
Chen 2015b	22	1862	11.82	[7.42; 17.83]	1.0%
Cheng 2013	9	2296	3.92	[1.79; 7.43]	0.9%
Cui 2012	43	1950	22.05	[16.00; 29.59]	1.0%
Fu 2010	41	3894	10.53	[7.57; 14.26]	1.0%
Fu 2011	215	11631	18.49	[16.12; 21.10]	1.1%
Gao 2014	126	3851	32.72	[27.33; 38.83]	1.1%
Gao 2015a	25	791	31.61	[20.56; 46.30]	1.0%
Gao 2015b	813	91448	8.89	[8.29; 9.52]	1.1%
He 2015	13	1017	12.78	[6.82; 21.76]	0.9%
Hou 2015	59	3899	15.13	[11.54; 19.48]	1.1%
Huang 2012	22	1335	16.48	[10.36; 24.84]	1.0%
Huang 2013	40	1696	23.58	[16.90; 31.98]	1.0%
Huang 2014	29	2715	10.68	[7.16; 15.30]	1.0%
Huang 2015a	38	2481	15.32	[10.86; 20.96]	1.0%
Huang 2015b	97	1813	53.50	[43.60; 64.88]	1.1%
Jia 2015	4	331	12.08	[3.30; 30.65]	0.7%
Jiang 2015	112	6071	18.45	[15.21; 22.16]	1.1%
Kong 2012	33	1142	28.90	[19.97; 40.34]	1.0%
Li 2011a	8	122	65.57	[28.73; 125.12]	0.9%
Li 2011b	6	288	20.83	[7.68; 44.79]	0.8%
Li 2012	17	182	93.41	[55.36; 145.34]	1.0%
Li 2013a	201	3900	51.54	[44.81; 58.95]	1.1%
Li 2014c	36	1114	32.32	[22.73; 44.46]	1.0%
Li 2015a	13	4768	2.73	[1.45; 4.66]	0.9%
Li 2015c	27	681	39.65	[26.29; 57.16]	1.0%
Lin 2015	14	712	19.66	[10.79; 32.77]	1.0%
Liu 2011	20	574	34.84	[21.41; 53.30]	1.0%
Liu 2013b	18	1082	16.64	[9.89; 26.16]	1.0%
Liu 2014	22	2173	10.12	[6.36; 15.29]	1.0%
Liu 2015a	33	1619	20.38	[14.07; 28.51]	1.0%
Liu 2015b	139	12935	10.75	[9.04; 12.68]	1.1%
Liu 2015c	8	657	12.18	[5.27; 23.85]	0.9%
Mai 2013	4	480	8.33	[2.28; 21.20]	0.7%
Mao 2012	3863	237216	16.28	[15.78; 16.80]	1.1%
Ming 2012	23	580	39.66	[25.30; 58.91]	1.0%
Pan 2013a	28	689	40.64	[27.17; 58.20]	1.0%
Qian 2015	24	2144	11.19	[7.19; 16.61]	1.0%
Qu 2011	0	54	0.00	[0.00; 66.03]	0.2%
Ren 2012	4	1378	2.90	[0.79; 7.42]	0.7%
Ren 2015	70	9680	7.23	[5.64; 9.13]	1.1%
Sheng 2015	39	1172	33.28	[23.77; 45.21]	1.0%
Shu 2015a	18	190	94.74	[57.12; 145.59]	1.0%
Shu 2015b	20	480	41.67	[25.63; 63.62]	1.0%
Song 2013	86	1834	46.89	[37.68; 57.59]	1.1%
Song 2015	66	1458	45.27	[35.18; 57.23]	1.1%
Sun 2015	1	341	2.93	[0.07; 16.23]	0.3%
Tang 2012a	1	7	142.86	[3.61; 578.72]	0.4%
Tang 2015	9	264	34.09	[15.70; 63.73]	0.9%
Wang 2011a	90	6029	14.93	[12.02; 18.32]	1.1%
Wang 2011b	16	658	24.32	[13.96; 39.19]	1.0%
Wang 2013a	24	1634	14.69	[9.43; 21.78]	1.0%
Wang 2014a	9	367	24.52	[11.27; 46.04]	0.9%
Wang 2014c	5	134	37.31	[12.22; 84.93]	0.8%
Wang 2015a	97	4158	23.33	[18.96; 28.39]	1.1%
Wang 2015c	11	3332	3.30	[1.65; 5.90]	0.9%
Wang 2015d	211	958	220.25	[194.38; 247.85]	1.1%
Wei 2013	296	6028	49.10	[43.79; 54.86]	1.1%
Wu 2012	25	465	53.76	[35.09; 78.35]	1.0%
Wu 2013	243	12338	19.70	[17.32; 22.30]	1.1%
Wu 2015	53	1772	29.91	[22.48; 38.94]	1.1%
Xie 2014	868	30035	28.90	[27.03; 30.86]	1.1%
Xing 2012	7	153	45.75	[18.59; 91.99]	0.8%
Xing 2013	4	130	30.77	[8.45; 76.91]	0.7%
Xu 2014a	51	3572	14.28	[10.65; 18.73]	1.1%
Xue 2014	48	1237	38.80	[28.75; 51.12]	1.1%
Yang 2012	45	1809	24.88	[18.20; 33.15]	1.0%
Yang 2013	12	436	27.52	[14.30; 47.58]	0.9%
Yang 2014c	18	1435	12.54	[7.45; 19.75]	1.0%
Yang 2015a	44	3384	13.00	[9.46; 17.42]	1.0%
Yang 2015b	21	725	28.97	[18.02; 43.94]	1.0%
Yao 2015	63	3310	19.03	[14.66; 24.29]	1.1%
Ye 2014	87	2457	35.41	[28.46; 43.50]	1.1%
Yu 2014	16	576	27.78	[15.96; 44.72]	1.0%
Yu 2015a	18	1233	14.60	[8.67; 22.97]	1.0%
Yu 2015b	20	534	37.45	[23.02; 57.25]	1.0%
Yu 2015c	265	17232	15.38	[13.59; 17.33]	1.1%
Yuan 2011	142	8702	16.32	[13.76; 19.21]	1.1%
Yue 2015	13	404	32.18	[17.24; 54.40]	0.9%
Zhang 2010a	481	39546	12.16	[11.11; 13.29]	1.1%
Zhang 2011b	51	1187	42.97	[32.16; 56.11]	1.1%
Zhang 2011c	31	1519	20.41	[13.91; 28.84]	1.0%
Zhang 2011d	41	3041	13.48	[9.69; 18.25]	1.0%
Zhang 2011f	51	1597	31.93	[23.87; 41.78]	1.1%
Zhang 2012b	31	1054	29.41	[20.07; 41.49]	1.0%
Zhang 2013b	33	775	42.58	[29.49; 59.28]	1.0%
Zhang 2014a	8	956	8.37	[3.62; 16.42]	0.9%
Zhang 2014b	22	1159	18.98	[11.93; 28.60]	1.0%
Zhang 2014c	3	966	3.11	[0.64; 9.05]	0.6%
Zhang 2015b	98	4604	21.29	[17.31; 25.88]	1.1%
Zhang 2015c	21	213	98.59	[62.07; 146.76]	1.0%
Zhao 2013	17	882	19.27	[11.27; 30.68]	1.0%
Zhao 2014b	17	2033	8.36	[4.88; 13.35]	1.0%
Zhao 2015	69	1034	66.73	[52.29; 83.70]	1.1%
Zhou 2013b	15	242	61.98	[35.10; 100.17]	1.0%
Zhu 2010	3	74	40.54	[8.44; 113.94]	0.6%
Zhu 2014a	29	6850	4.23	[2.84; 6.07]	1.0%
Zhu 2014b	79	2466	32.04	[25.44; 39.77]	1.1%
Zou 2013	268	7740	34.63	[30.66; 38.94]	1.1%
Zou 2015	48	2125	22.59	[16.70; 29.84]	1.1%
Zuo 2015	2	420	4.76	[0.58; 17.09]	0.5%
**Random effects model**		**672616**	**22.83**	**[19.88; 26.23]**	**100%**

Heterogeneity: I-squared=98%, p<0.0001

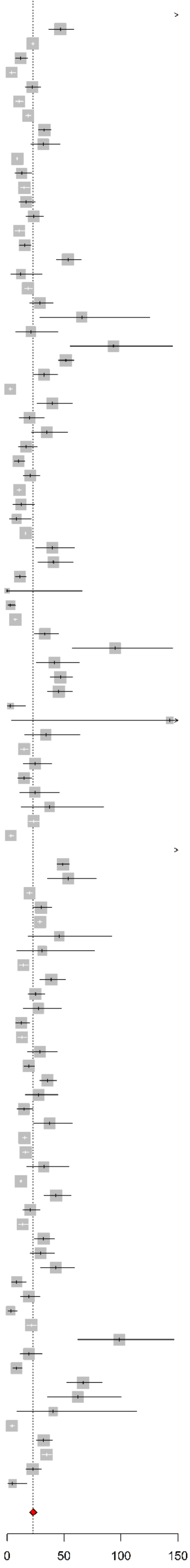

Figure S3. Forest plot of the incidence density (reported as patients per 1000 ventilator-days) of ventilator-associated pneumonia (VAP) using a random-effects model.
