# Supplementary material for: Incidence, temporal trend and factors associated with ventilator-associated pneumonia in mainland China: a systematic review and meta-analysis
Source: BMC Infect Dis. 2017 Jul 4;17:468. doi: 10.1186/s12879-017-2566-7 (PMC5496595; doi:10.1186/s12879-017-2566-7)
Supplement: Supplementary file 2 — Risk of bias assessment tool. (PDF 119 kb) [file 12879_2017_2566_MOESM2_ESM.pdf]

Additional File 2: Risk of bias assessment tool

Name of author(s)____________________ Year of publication____________________
Name of paper/study____

Note: if there is insufficient information in the article to permit a judgment for a particular item, please answer **No (High Risk of Bias)** for that particular item.

**Risk of Bias Item**	**Answer:**<br>**Yes** (Low Risk of Bias) **or** **No** (High risk of Bias)
**External Validity**	
1. Was the study's target population a close representation of the national population in relation to relevant variables, e.g. age, sex, occupation?	
2. Was the sampling frame a true or close representation of the target population?	
3. Was some form of random selection used to select the sample, OR, was a census undertaken?	
4. Was the likelihood of non-response bias minimal?	
**Internal Validity**	
5. Were data collected directly from the subjects (as opposed to a proxy)?	
6. Was an acceptable case definition of VAP used in the study?	
7. Was a reliable and valid diagnosis method for VAP utilized?	
8. Was the same mode of data collection used for all subjects?	
9. Was the length of the shortest follow-up period for VAP appropriate?	
10. Were the numerator(s) and denominator(s) for the calculation of the incidence of VAP appropriate?	

**Summary item on the overall risk of study bias**<br><br>**Low Risk of Bias:** 8 or more "yes" answers. Further research is very unlikely to change our confidence in the estimate.<br><br>**Moderate Risk of Bias:** 6 to 7 "yes" answers. Further research is likely to have an important impact on our confidence in the estimate and may change the estimate.<br><br>**High Risk of Bias:** 5 or fewer "yes" answers. Further research is very likely to have an important impact on our confidence in the estimate and is likely to change the estimate.	
